# Microorganisms and dissolved metabolites distinguish Florida's Coral Reef habitats

**DOI:** 10.1093/pnasnexus/pgad287

**Published:** 2023-09-05

**Authors:** Cynthia C Becker, Laura Weber, Brian Zgliczynski, Chris Sullivan, Stuart Sandin, Erinn Muller, Abigail S Clark, Melissa C Kido Soule, Krista Longnecker, Elizabeth B Kujawinski, Amy Apprill

**Affiliations:** Marine Chemistry and Geochemistry Department, Woods Hole Oceanographic Institution, Woods Hole, MA 02543, USA; Biological Oceanography, Massachusetts Institute of Technology-Woods Hole Oceanographic Institution Joint Program in Oceanography/Applied Ocean Science and Engineering, Cambridge, MA 02139, USA; Marine Chemistry and Geochemistry Department, Woods Hole Oceanographic Institution, Woods Hole, MA 02543, USA; Scripps Institution of Oceanography, University of California San Diego, La Jolla, CA 92093, USA; Scripps Institution of Oceanography, University of California San Diego, La Jolla, CA 92093, USA; Scripps Institution of Oceanography, University of California San Diego, La Jolla, CA 92093, USA; Elizabeth Moore International Center for Coral Reef Research and Restoration, Mote Marine Laboratory, Summerland Key, FL 33042, USA; Coral Health and Disease Program, Mote Marine Laboratory, Sarasota, FL 34236, USA; Elizabeth Moore International Center for Coral Reef Research and Restoration, Mote Marine Laboratory, Summerland Key, FL 33042, USA; Marine Science and Technology Department, The College of the Florida Keys, Key West, FL 33040, USA; Marine Chemistry and Geochemistry Department, Woods Hole Oceanographic Institution, Woods Hole, MA 02543, USA; Marine Chemistry and Geochemistry Department, Woods Hole Oceanographic Institution, Woods Hole, MA 02543, USA; Marine Chemistry and Geochemistry Department, Woods Hole Oceanographic Institution, Woods Hole, MA 02543, USA; Marine Chemistry and Geochemistry Department, Woods Hole Oceanographic Institution, Woods Hole, MA 02543, USA

**Keywords:** coral reefs, metagenomics, metabolomics, Florida's Coral Reef

## Abstract

As coral reef ecosystems experience unprecedented change, effective monitoring of reef features supports management, conservation, and intervention efforts. Omic techniques show promise in quantifying key components of reef ecosystems including dissolved metabolites and microorganisms that may serve as invisible sensors for reef ecosystem dynamics. Dissolved metabolites are released by reef organisms and transferred among microorganisms, acting as chemical currencies and contributing to nutrient cycling and signaling on reefs. Here, we applied four omic techniques (taxonomic microbiome via amplicon sequencing, functional microbiome via shotgun metagenomics, targeted metabolomics, and untargeted metabolomics) to waters overlying Florida's Coral Reef, as well as microbiome profiling on individual coral colonies from these reefs to understand how microbes and dissolved metabolites reflect biogeographical, benthic, and nutrient properties of this 500-km barrier reef. We show that the microbial and metabolite omic approaches each differentiated reef habitats based on geographic zone. Further, seawater microbiome profiling and targeted metabolomics were significantly related to more reef habitat characteristics, such as amount of hard and soft coral, compared to metagenomic sequencing and untargeted metabolomics. Across five coral species, microbiomes were also significantly related to reef zone, followed by species and disease status, suggesting that the geographic water circulation patterns in Florida also impact the microbiomes of reef builders. A combination of differential abundance and indicator species analyses revealed metabolite and microbial signatures of specific reef zones, which demonstrates the utility of these techniques to provide new insights into reef microbial and metabolite features that reflect broader ecosystem processes.

**Significance Statement**

Microorganisms and the dissolved metabolites they process are central to the functioning of ocean ecosystems. These “invisible” ocean features are poorly understood in biodiverse and productive coral reef ecosystems, where they contribute to nutrient cycling and signaling cues among reef organisms. This study demonstrates that reef water microbes and metabolites successfully distinguish reef habitats, including geographical, reef compositional, and environmental features, and together they provide novel insights into reef ecosystem characteristics. Microbes and dissolved metabolites offer a new means to examine reef features and have applications for conservation, monitoring, and restoration efforts in these changing ecosystems.

## Introduction

Coral reefs, one of the most biodiverse and economically valuable ocean ecosystems, are dynamic habitats that are impacted worldwide by climate change and other human pressures. Coral reef ecosystems have responded positively and negatively to disturbances through time (1). In the past 50 years, however, hard coral cover, a metric that represents the percentage of benthic habitat these keystone species encompass, has been declining worldwide. This includes 20–30% losses in Caribbean reefs (2, 3), 50% in the Great Barrier Reef (4), and as high as 92% losses in Florida's Coral Reef (FCR) (5–7). Although reef environments are declining, we are only beginning to understand the value of the innumerable microbial and chemical components of coral reefs that interact to support the most biodiverse ocean ecosystem.

Central to the success of reef ecosystems are the invisible microbial and chemical dynamics that play a major role in reef processes, such as settlement (8–10) and coral growth and health (11–15). Detrimental microbial and chemical dynamics may even be central to reef decline, as exemplified by the microbialization hypothesis, in which algal-exuded dissolved organic carbon promotes growth of pathogenic microorganisms, which in turn threaten corals and other reef organisms (16). Although microorganisms and chemicals are emerging as critical components for the success or failure of reefs, monitoring programs generally do not incorporate these parameters, due to the lack of scientific evidence documenting their utility as well as the high levels of cost and expertise needed to evaluate them. Therefore, integrative studies that employ a suite of chemical and microbial comparisons are critical for understanding how these parameters may differentiate reef habitats and reflect ecological dynamics.

Reef microorganisms and dissolved chemicals, or metabolites, are well-poised to serve as sensors of reef ecosystem dynamics (17, 18). The production of dissolved metabolites within reef seawater is species-specific, with corals producing diverse nitrogen and phosphorus-based compounds and macroalgae yielding prenol lipids, steroids (19), and organic compounds enriched in neutral sugars (20). Additionally, coral host metabolomes are further influenced by metabolites derived from algal symbionts (21). Collectively, these organism-specific signals make up the metabolome signature of a reef, which differs from open-ocean waters (22). In seawater overlying a reef, microorganisms are a primary consumer of metabolites, and while certain lineages of microorganisms are enriched in response to species-specific benthic exudates (20, 23, 24), we are only beginning to understand which individual metabolites may be responsible for such enrichment (25). Studies have documented relationships between reef microbial composition and reef parameters, including hard coral cover (26, 27), temperature (28, 29), chlorophyll (28), organic carbon (29), biogeography (27, 30), and reef protection (31). To date, most investigations study either metabolites or microbes in isolation (16, 19, 22), though studies are increasingly combining techniques (32, 33). A direct comparison of reef water metabolites and microorganisms is needed to inform the individual or collective value of these factors within reef habitats.

FCR is an ideal environment for examining reef microbial and metabolic characteristics within the context of broader reef processes. Decades of research have demonstrated biogeographic reef zones partitioning the system, largely driven by reef hydrography, with the Gulf of Mexico Loop Current and meandering Florida Current producing many gyres and eddies (34–36). These currents interact with tidal mixing from Florida Bay, especially in the Lower and Middle Keys, but less so in the Upper Keys, which are more isolated from Florida Bay water, contributing to zonal patterns of nearshore nutrients and organic matter (37). For example, the Upper, Middle, and Lower Keys zones vary and are distinct from more offshore reef zones, such as the Marquesas and Dry Tortugas, in terms of, but not limited to, total organic carbon (TOC), nutrients, and turbidity (38). Geological evidence also reflects these regional patterns, with historical reef accretion and present-day coral calcification rates higher in the remote Dry Tortugas zone compared to other zones within the Florida Keys (39, 40). Presently, FCR coral communities are facing the highly contagious stony coral tissue loss disease (SCTLD), a devastating disease that has already led to the regional extinction of the pillar coral, *Dendrogyra cylindrus*, in Florida (41, 42). This years-long disease outbreak is likely caused by a bacterial pathogen(s) (43–45), and although microbial and metabolomic indicators have been identified (21, 46–50), the causative agent remains elusive. This disease, along with climate pressures causing unprecedented levels of warming leading to bleaching and mortality (51), is leading to increased homogenization on the reefs (52), though some zonation within the benthic community persists (53, 54). Considering the recent stressors to the FCR, especially SCTLD, examinations of microorganisms and metabolites have the potential to add significant value to the breadth of historic and present research on FCR reef ecology.

To better understand the metabolite and microbial landscape of FCR and the potential utility of these invisible sensors to reef ecosystem dynamics, we leveraged a cruise of opportunity on OceanX's M/V *Alucia* during June 2019 to study 85 reefs dispersed along the 500-km reef tract. We examined if and how reef water microbes and metabolites differentiate each reef within the context of geography, reef benthos, disease, and environmental parameters, using four omic approaches (taxonomic microbiome via amplicon sequencing, functional microbiome via shotgun metagenomics, targeted metabolomics, and untargeted metabolomics). We also conducted microbiome profiling via amplicon sequencing of near-coral seawater and coral hosts. Our results demonstrate that microbes and metabolites successfully distinguish between individual reefs according to biogeochemical zones and that their combined use provides novel insights into reef ecosystem parameters that may benefit monitoring, conservation, and restoration activities in these important ecosystems.

## Results

**Fig. 1.**
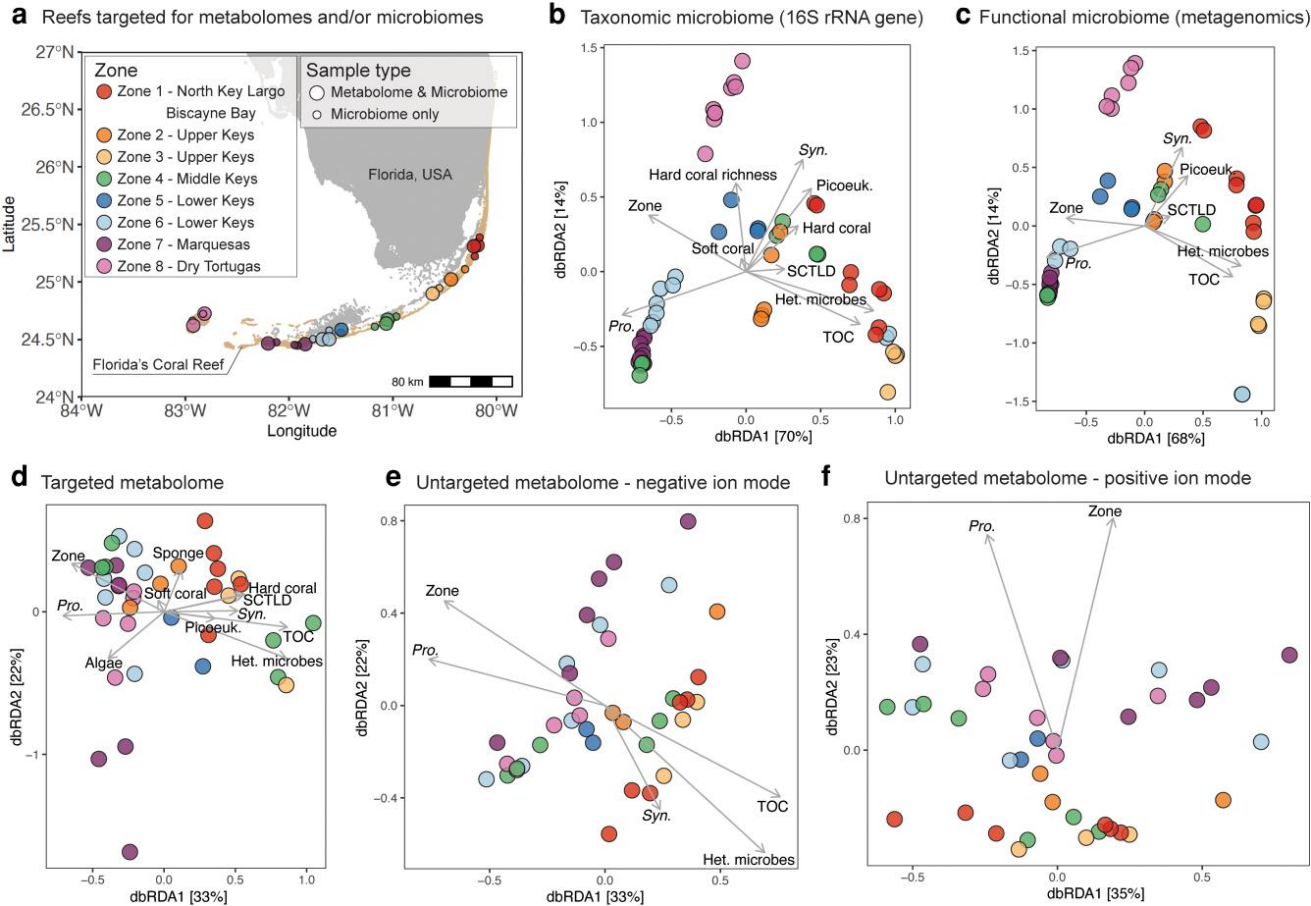
dbRDA reveals seawater microbiomes and metabolomes are significantly explained by Florida's Coral Reef biogeography and measured reef microbial and environmental parameters (ANOVA on dbRDA model, *P* < 0.05). (a) Map of 27 coral reefs (out of 85 reefs visited in total) sampled across eight zones for either both seawater metabolomes and microbiomes (13 reefs) or only seawater microbiomes (27 reefs total) during June 2019. The dbRDA include (b) reef water taxonomic microbiome via 16S rRNA gene sequencing of bacteria and archaea, (c) functional microbiome via shotgun metagenomics, (d) targeted metabolomes, (e) untargeted metabolomes that ionized in negative mode, and (f) untargeted metabolomes that ionized in positive mode. *Syn*., *Synechococcus*; *Pro*., *Prochlorococcus*; Picoeuk., picoeukaryotes; Het. Microbes, heterotrophic microbes.

**Fig. 2.**
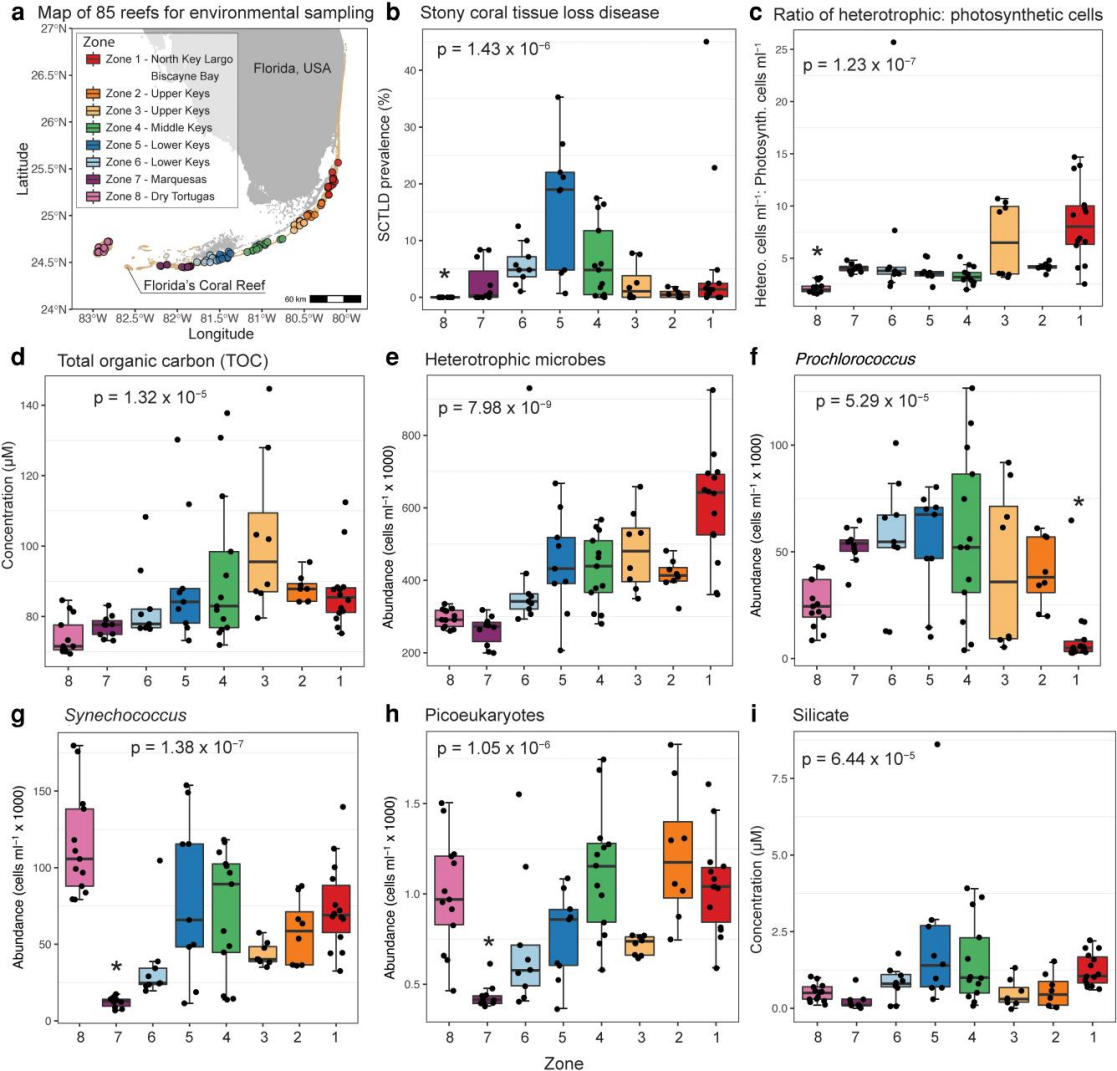
Zones in FCR have significantly different disease prevalence, microbial abundances, and organic carbon and silicate concentrations. (a) Map of all 85 reefs sampled for environmental variables are oriented by zone. Environmental variables that changed significantly by zone include (b) SCTLD prevalence (percent of live coral), (c) ratio of heterotrophic to photosynthetic microbial cells, (d) total organic carbon concentration, (e) heterotrophic microbial abundances (unpigmented bacteria and archaea), (f) *Prochlorococcus* abundances, (g) *Synechococcus* abundances, (h) picoeukaryote abundances, and (i) silicate concentration that was significantly different between FCR zones as a result of a Kruskal–Wallis test and less than the Bonferroni-corrected *P*-value of 0.00192. Asterisks denote the zone was significantly different than all other zones tested in the pairwise Wilcoxon rank sum test (Benjamini–Hochberg adjusted *P* < 0.05).

This comparative omic study was conducted over 15 days in June 2019 along FCR. At 13 geographically dispersed reefs, we collected seawater (1.7 L in biological triplicates) from within 0.5 m of the reef benthos for both targeted and untargeted metabolome analyses (Fig. 1a). At 27 reefs, including the 13 examined for metabolomics, we collected benthic seawater (2 L in duplicate) for taxonomic (bacteria and archaea 16S ribosomal RNA gene) and functional (shotgun metagenome) microbiome analyses and chlorophyll (Fig. 1a). Given the active SCTLD outbreak, we also targeted apparently healthy and diseased coral tissue and near-coral seawater within 1–3 cm from the coral for taxonomic microbiome (16S rRNA gene) analysis (11 reefs). To comprehensively assess the reef habitat, at 85 reefs (Fig. 2a), we measured prevalence of SCTLD, nutrients (TOC, total organic nitrogen [TON], and inorganic nutrients), and abundances of microbial functional groups (*Prochlorococcus*, *Synechococcus*, picoeukaryotes, and heterotrophic microbes [unpigmented bacteria and archaea]), from reef depth waters (Fig. 2a). Of the 85 reefs, high-resolution photomosaics were taken at 45 reefs to examine the composition of benthic organisms (Fig. S1). Detailed methods and information on these data sets can be found in the Methods S02.

Four high-resolution omic data sets were produced from the benthic seawater samples. The reef water taxonomic microbiome (27 reefs) approach yielded 43,118 ± 6,834 sequences per sample and 468 total amplicon sequence variants (ASVs, similar to microbial species-level designations (55)), with 110–191 ASVs per sample (see Methods S02). The functional microbiome (metagenomics, 27 reefs) effort produced 13.6 ± 3.4 M paired-end reads per sample, which were assembled into contigs and annotated and mapped to 592,912 bacterial gene annotations. Targeted metabolomics (13 reefs) was applied to the reef water, with samples analyzed via ultrahigh-performance liquid chromatography coupled to a heated electrospray ionization source (H-ESI) and triple stage quadrupole mass spectrometer. This approach quantified 39 environmentally relevant dissolved metabolites in the samples. The untargeted metabolomic approach analyzed extracts via ultrahigh-performance liquid chromatography system coupled to an Orbitrap Fusion Lumos Tribrid mass spectrometer and ionized chemical features in two modes, positive and negative (see Methods S02). After removing features associated with blanks and with low variance across samples, 2,759 features remained in the positive ion mode data set, and 1,428 features remained in the negative ion mode data set.

### Microorganism and metabolite compositions reflect zonal habitat patterns

Analysis of seawater omic data sets (taxonomic and functional microbiome and targeted and untargeted metabolome) via ordinations revealed significant clustering of all data types according to biogeographic reef zone (depicted via colors in Fig. 1). A distance-based redundancy analysis (dbRDA) followed by an ANOVA revealed that biogeographic reef zone was the most significant parameter in structuring the composition of seawater omic data sets (Table 1; Pseudo-F values indicate significance). For untargeted metabolomes with triplicate biological replication per reef, reef structured the chemical composition to a greater extent than zone, which contained multiple reefs, though both were significant (Fig. S2; permutational multivariate ANOVA [PERMANOVA] results in Table S1).

**Table 1.** Reef benthic and environmental characteristics significantly explained reef seawater omics.

	Microbiome						Metabolome								
	Taxonomic (16S rRNA gene)			Functional (metagenomics)			Targeted			Untargeted negative			Untargeted positive		
PERMANOVA^a^ term	*F* ^b^	*R* ^2c^	*P*-value	*F* ^b^	*R* ^2c^	*P*-value	*F* ^b^	*R* ^2c^	*P*-value	*F* ^b^	*R* ^2c^	*P*-value	*F* ^b^	*R* ^2c^	*P*-value
Zone	97.0	0.126	**0.001**	62.5	0.167	**0**.**001**	6.21	0.018	**0**.**001**	6.02	0.021	**0**.**002**	3.38	0.020	**0**.**012**
*Prochlorococcus*	49.2	0.095	**0**.**001**	33.6	0.120	**0**.**001**	3.10	0.009	**0**.**001**	4.31	0.054	**0**.**002**	4.06	0.054	**0**.**008**
Total organic carbon	60.1	0.132	**0**.**001**	39.4	0.128	**0**.**001**	2.90	0.035	**0**.**002**	2.25	0.020	**0**.**03**	1.36	0.006	0.185
Heterotrophic microbes	34.3	0.187	**0**.**001**	19.0	0.156	**0**.**001**	3.23	0.073	**0**.**002**	2.24	0.060	**0**.**044**	1.79	0.062	0.099
*Synechococcus*	21.0	0.040	**0**.**001**	12.2	0.039	**0**.**001**	1.99	0.006	**0**.**024**	2.40	−0.006	**0**.**028**	2.04	−0.008	0.07
SCTLD prevalence	5.50	0.003	**0**.**007**	3.28	0.005	**0**.**034**	3.53	0.014	**0**.**001**	1.57	0.000	0.126	0.95	−0.010	0.402
Picoeukaryotes	4.57	0.011	**0**.**007**	3.76	0.010	**0**.**018**	2.30	0.005	**0**.**014**	1.20	0.002	0.262	0.97	0.001	0.379
Soft coral cover	3.17	0.010	**0**.**043**	2.76	0.001	0.07	2.73	0.016	**0**.**002**	1.37	0.007	0.174	1.11	−0.007	0.275
Hard coral cover	4.26	0.012	**0**.**017**	2.37	−0.001	0.091	1.95	0.044	**0**.**031**	1.35	0.017	0.205	0.91	−0.003	0.434
Sponge cover	2.89	0.090	0.052	2.49	0.089	0.075	1.98	−0.001	**0**.**034**	0.69	−0.009	0.68	0.81	−0.013	0.512
Algae (fleshy and turf) cover	1.18	0.024	0.292	1.53	0.003	0.176	2.16	0.035	**0**.**02**	1.60	0.000	0.112	1.25	−0.010	0.217
Hard coral richness	2.87	0.075	**0**.**047**	1.69	0.092	0.174	1.51	0.025	0.121	1.65	0.027	0.11	1.11	0.009	0.264

Bold indicates significant at *P* < 0.05.

a PERMANOVA is a permutational ANOVA for distance-based redundancy analysis (dbRDA) using 999 permutations.

b Pseudo-*F* value indicates overall significance of the term (higher is more significant).

c Adjusted *R*^2^ was calculated using the varpart function and represents the amount of variance explained by the individual fraction (variable), and this can be negative because the underlying dbRDA can yield negative eigenvalues.

We further demonstrated that benthic, geographic, and environmental parameters significantly structured the seawater microbiome and metabolome compositions (PERMANOVA for dbRDA with Bray–Curtis dissimilarity, *P* < 0.05; Fig. 1 and Table 1). Reef zone and *Prochlorococcus* abundance structured all omic data types. In the microbiome analyses, zone and *Prochlorococcus* explained between 12.6–16.7% and 9.5–12% of the variance, respectively, and the variance explained was lower in the metabolome analyses (Table 1, adjusted *R*^2^). TOC, heterotrophic microbes, and *Synechococcus* explained changes in the microbiome as well as in the targeted and negative mode untargeted metabolome, with heterotrophic microbial abundances explaining the most variation (15.6–18.7% in microbiome analyses and 6.0–7.3% in metabolome analyses) (Fig. 1 and Table 1). Significant structuring by other parameters varied by omic data type, with the targeted metabolome significantly structured by all tested parameters (Fig. 1 and Table 1).

Dry Tortugas National Park (zone 8) was distinctly clustered from zone 1 to zone 7 in seawater taxonomic and functional microbiomes (Fig. 1b and c). Environmental parameters were also different at Dry Tortugas, which harbored significantly lower prevalence of SCTLD, seawater TOC, heterotrophic microbial abundances, ratio of heterotrophic microbes:photosynthetic microbes, and cover of turf algae compared to zones 1–7. In contrast, *Synechococcus* abundances were higher at zone 8 compared to other zones (Wilcoxon rank sum test with Bonferroni-corrected *P* < 0.001667; Fig. S3).

Benthic components significantly explained differences in both the targeted metabolomes (soft coral, hard coral, sponge, and algae cover) and taxonomic microbiomes (soft and hard coral cover and hard coral richness) (Fig. 1b and d and Table 1). Unlike reef seawater omic data, benthic reef composition was not significantly structured by zone (PERMANOVA on principal components analysis; Fig. S1b). Individual tests of zonal changes in hard coral and algal cover, as well as hard coral species richness, common metrics used to assess reef quality and coral diversity, changed slightly but were not significantly different (Kruskal–Wallis rank sum test with Bonferroni-corrected *P* > 0.00192; Figs. S4 and S5). In contrast, hard coral species composition alone was significantly different by zone, with Dry Tortugas National Park (zone 8) clustered separately from other reefs (PERMANOVA, *P* < 0.05; Fig. S5). Distance to shore and percent cover of algae were both significantly negatively correlated with coral cover (linear regression, *P* < 0.05; Figs. S6 and S7).

Zonal changes were evident in many environmental parameters measured at 85 reefs across eight zones (Kruskal–Wallis test with Bonferroni-corrected *P* < 0.00192; Fig. 2). The ratio of heterotrophic microbes:photosynthetic microbes, TOC concentrations, and heterotrophic microbial abundances all increased northeastward across reefs from zone 8 to zone 1 (Fig. 2c–e). SCTLD prevalence generally peaked in zones 4–6 (Fig. 2b), zones that also had high silicate concentrations (Fig. 2i). Across zones, abundances of *Prochlorococcus* had a parabolic distribution, while *Synechococcus* and picoeukaryote abundances were higher in Dry Tortugas and then increased from the Marquesas zone 7 to zone 1 (Fig. 2f–h). Chlorophyll *a* and inorganic nitrogen and phosphorus concentrations shifted with zone, but these trends were not significant (Kruskal–Wallis test with Bonferroni-corrected *P* > 0.00192; Fig. S8).

### Reef seawater metabolites driven by FCR zone

**Fig. 3.**
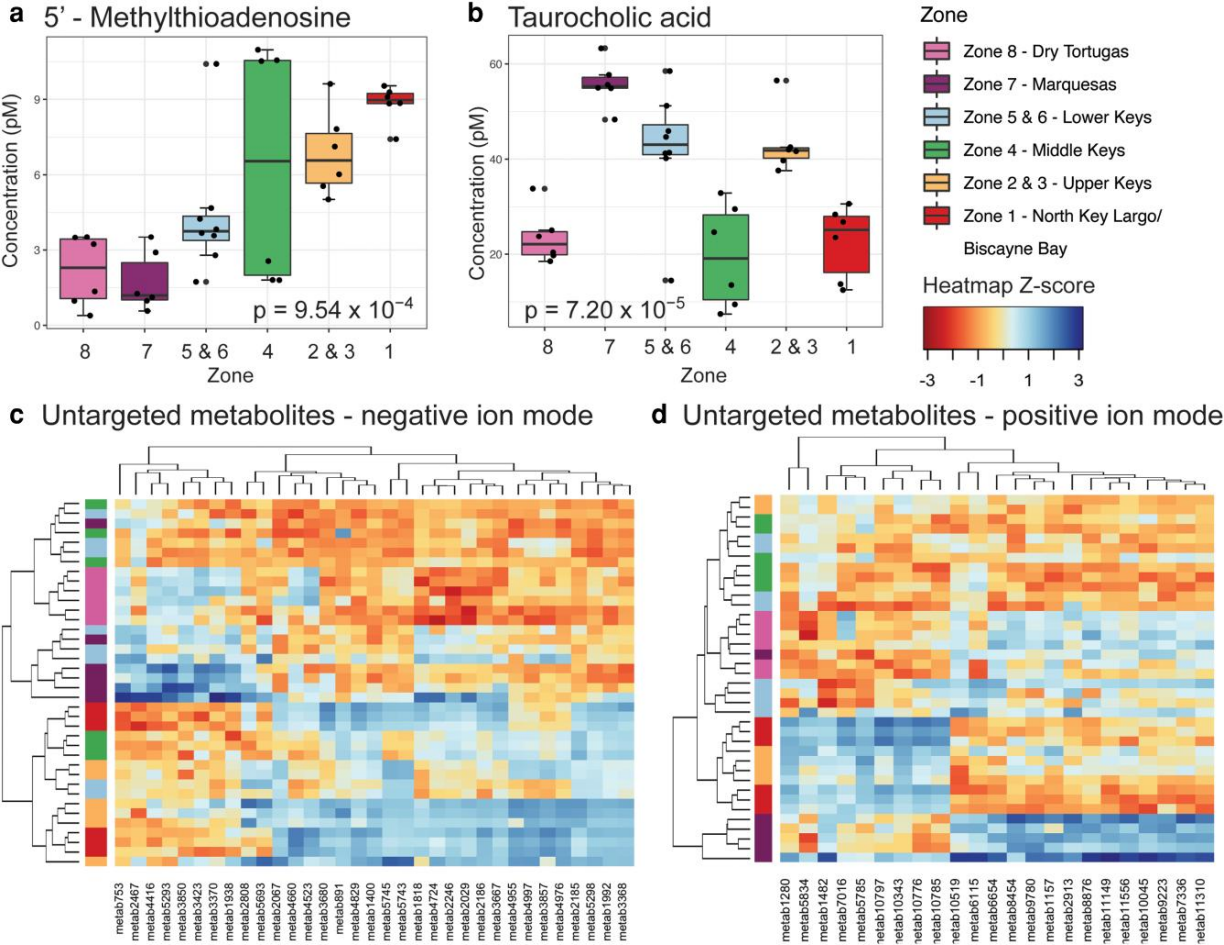
Zones in FCR harbor significantly different concentrations of dissolved metabolites (a) MTA and (b) taurocholic acid. Concentrations were significantly different between FCR zones (Kruskal–Wallis test, Bonferroni-corrected *P* < 0.00128). Heat maps of *z*-score standardized feature peak intensities depict untargeted metabolites that ionized in (c) negative or (d) positive ion modes that were significantly different across zones (ANOVA or Kruskal–Wallis test, significant at Bonferroni-corrected *P*; see Methods S02). Squares to the left of the heat map indicate reef zone. Adjacent zones 5 and 6 in the Lower Keys and zones 2 and 3 in the Upper Keys were combined to ensure at least two reefs were within each group for statistical tests.

To evaluate which metabolites may be signatures of reef zones, we conducted ANOVA or Kruskal–Wallis tests on targeted and untargeted (positive and negative ion modes) metabolites. Two of the 39 targeted metabolites in reef seawater changed across reef zones: 5′-methylthioadenosine (MTA) and taurocholic acid (Kruskal–Wallis test with Bonferroni-corrected *P* < 0.00128; Fig. 3a and b). Taurocholic acid median concentrations were variable across zones, while MTA concentrations generally increased as sampling moved northeast (Fig. 3a and b). Additionally, the extracellular seawater concentration of MTA was positively related to the relative abundance of microbial genes found in seawater metagenomes that use MTA (sinks—MTA phosphorylase, MTA/S-adenosylhomocysteine [SAM] nucleosidase, and MTA/SAM deaminase) and produce MTA (sources—polyamine aminopropyltransferase and isovaleryl-homoserine lactone synthase) (model II ordinary least squares linear regression *P* < 0.001, 999 permutations; Fig. S9). Within the untargeted metabolome data sets, 33 of the 2,759 features in negative ion mode and 23 of the 1,428 features in positive ion mode differed across zones (Kruskal–Wallis test with Bonferroni-corrected *P* < 7.246 × 10^−5^ and *P* < 3.378 × 10^−5^ for negative and positive metabolites, respectively) (Fig. 3c and d).

### Reef seawater microbiome indicators of FCR zone

**Fig. 4.**
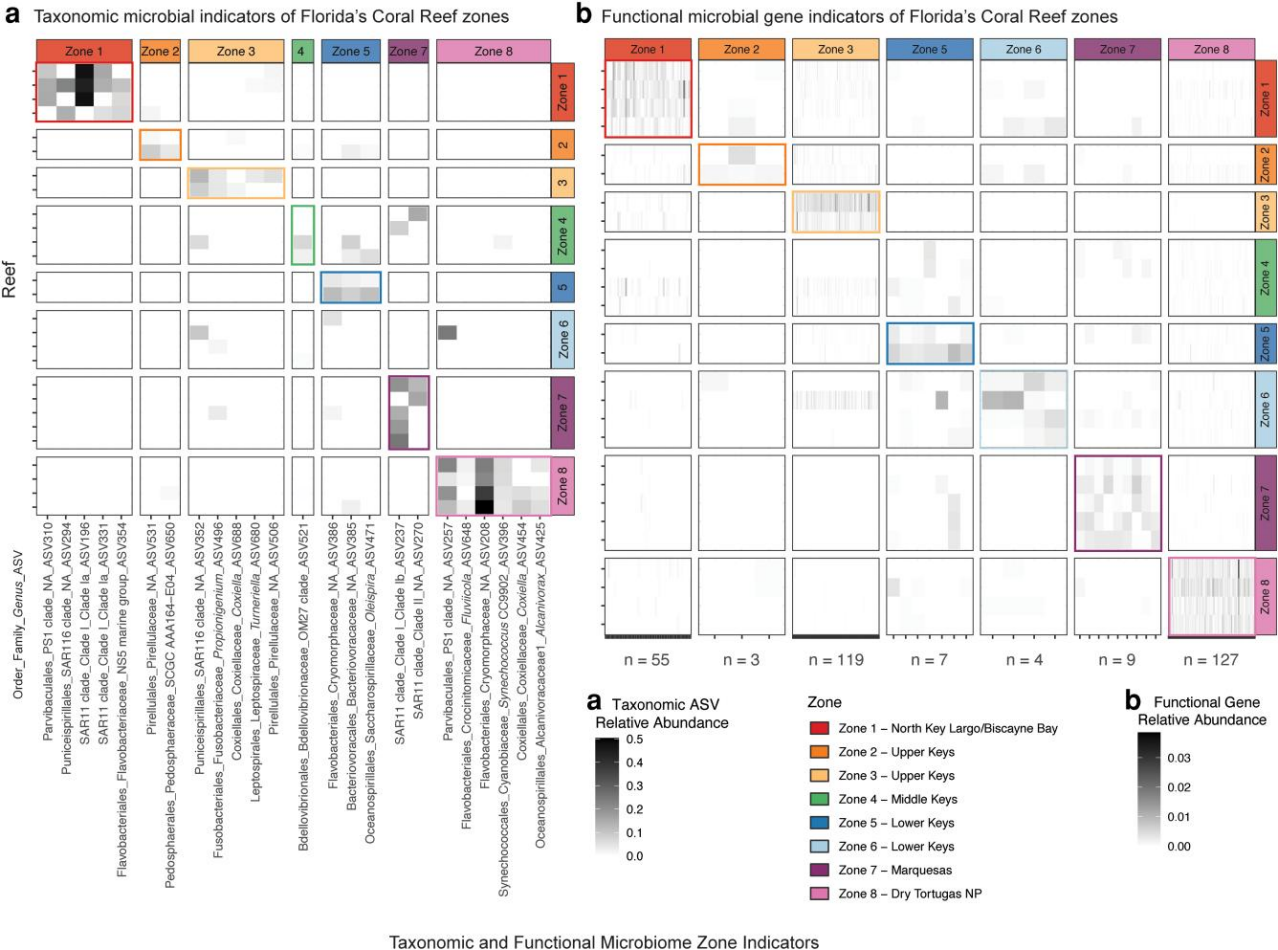
Indicator microbial taxa and functional genes across FCR zones. (a) Relative abundance across all reefs of ASVs diagnostic of individual reef zones and ASV identifier on the *x*-axis. (b) Relative abundance across all reefs of bacterial functional genes diagnostic of individual reef zones. Number of functional genes indicative in each grouping labeled on *x*-axis, with full summary of genes in File S04. All indicator taxa and functional genes determined by indicator value analysis (*A* ≥ 0.6, *B* ≥ 0.6, and *P* < 0.05). Taxa and functional genes are grouped by the associated zone they are indicative of.

We identified microbial ASVs and functional genes diagnostic of individual reef zones via indicator value analysis (indicspecies R package, specificity ≥0.6, sensitivity ≥0.6, *P* < 0.05; see Methods S02) (Fig. 4). Of the 468 seawater ASVs, 24 were diagnostic of one of the reef zones (summarized in File S04), with most representing Dry Tortugas (zone 8), including *Alcanivorax*, *Coxiella*, *Synechococcus*, *Fluviicola*, and the families Cryomorphaceae and PS1 clade of Parvibaculales (Fig. 4a; File S04). Zones 1 and 3 also harbored several indicator taxa, including SAR11 and SAR116 clades; the NS5 marine group and the PS1 clade of Parvibaculales in zone 1 and the SAR116 clade; and Pirellulaceae, *Propionigenium*, *Coxiella*, and *Turmeriella* (Fig. 4a) in zone 3. Zone 5, which harbored the greatest prevalence of SCTLD, had three indicator taxa including *Oleispira* and the families Cryomorphaceae and Bacteriovoracaceae.

Functional microbial genes were abundance-filtered to 103,665 genes (see Methods S02), and of these, 324 were diagnostic of a reef zone (Fig. 4b). All genes and their respective pathways are summarized in File S04. Like the taxonomic microbiome, the highest number of indicator genes were for Dry Tortugas National Park (zone 8, *n* = 127). Some of these genes were involved in biosynthetic pathways unique to zone 8, including arginine, cysteine, folate, glutamine, isoprenoid, serine, and ubiquinone biosynthesis. Additionally, genes from two types of ATP synthases (FoF1 and A/V) and 66 genes with unknown pathways were zone 8 indicators (Fig. 4b; File S04). Zone 3 in the Upper Keys also had many functional indicators (119), including some involved in asparagine, biotin, heme, lipoate, lysine, menaquinone, and nicotinamide adenine dinucleotide (NAD) biosynthesis pathways. Additionally, 75 of the 119 zone 3 indicator genes were from unknown pathways encoded for proteins such as a flagellar basal body protein, nitrate reductase, phosphate import protein, zinc metalloprotease, and a type II secretion system protein, among others (Fig. 4b; File S04). Zone 1 had 55 indicator genes, including a lipid A biosynthesis gene, as well as 30 indicator genes of unknown pathways (Fig. 4b; File S04). The other zones (2, 5, 6, and 7) harbored fewer indicator functional genes overall, and of these, zone 5 contained 7 indicators, including a protein in a pyrimidine salvage pathway (Fig. 4b; File S04).

### Coral host-associated microbiomes driven by reef habitat

**Fig. 5.**
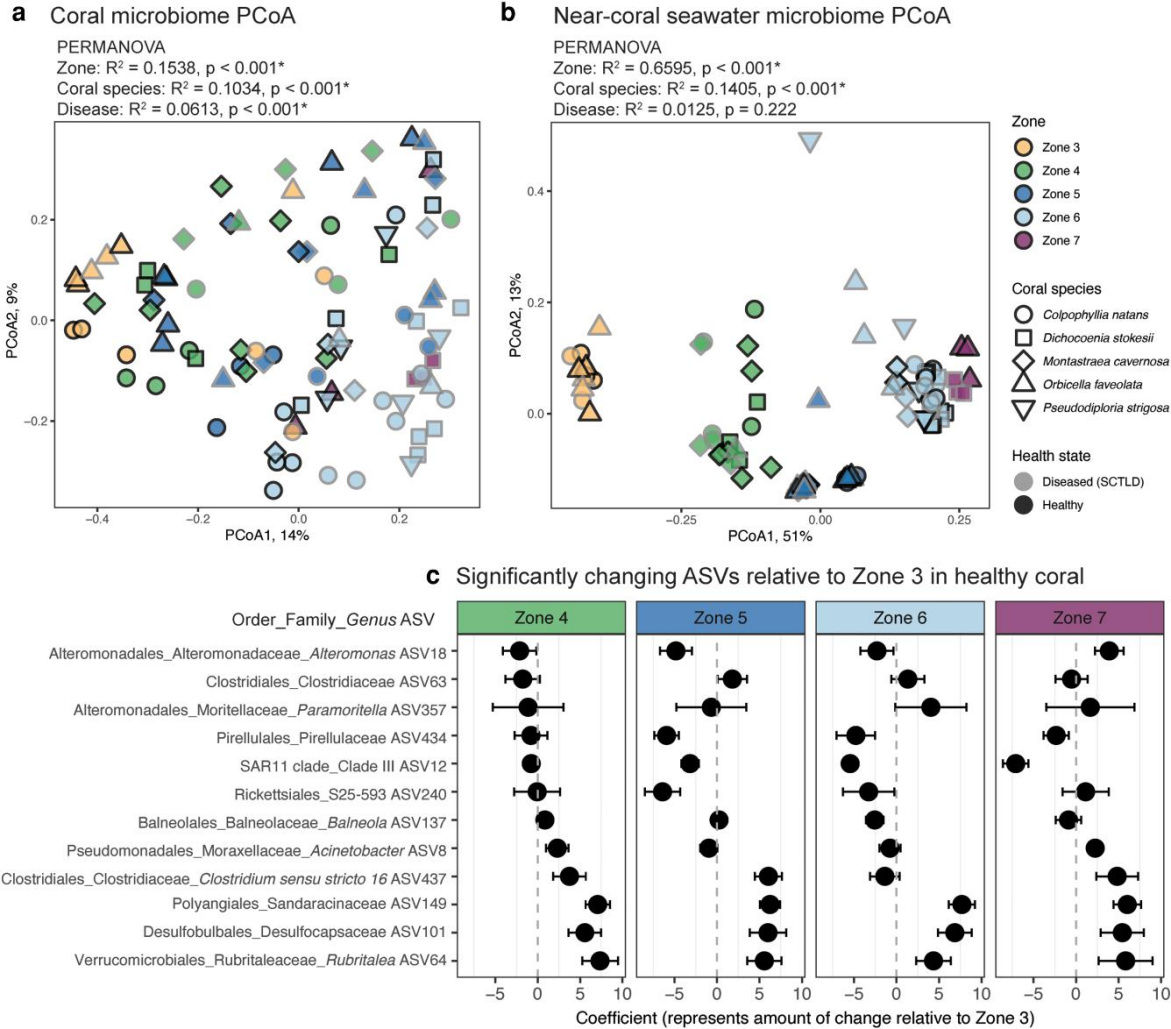
Coral and near-coral seawater taxonomic microbiomes are influenced by biogeography. Principal coordinates analysis (PCoA) of (a) coral and (b) near-coral seawater taxonomic microbiome beta diversity (Bray–Curtis dissimilarity). Results from a permutational ANOVA (PERMANOVA) displayed above each graph indicate coral microbiomes are significantly different across zones, coral species, and between apparently healthy and diseased corals, while near-coral seawater microbiomes are significantly different across zones and coral species (*P* < 0.05). Symbol outlines denote health state. (c) 12 ASVs significantly differed relative to zone 3, within apparently healthy coral microbiomes as identified by differential abundance tests (Benjamini–Hochberg adjusted *P* < 0.05; see Methods S02).

To examine if reef seawater zonal patterns are reflected in the corals, we applied the most cost-effective omic technique, taxonomic microbiome profiling, to corals and near-coral seawater collected within five reef zones (11 reefs). We collected samples from five coral species (*Colpophyllia natans*, *Dichocoenia stokesii*, *Montastraea cavernosa*, *Orbicella faveolata*, and *Pseudodiploria strigosa*) that were either apparently healthy or showing active SCTLD lesions, and this resulted in 116,942 ± 59,302 sequences per sample and a total of 3,020 ASVs after filtering (see Methods S02). Within coral-associated microbiomes, zone significantly structured the microbial community composition, reflective of microbial patterns in overlying seawater microbiomes (Figs. 5a and 1b). Coral species and disease state were also significantly related (PERMANOVA on Bray–Curtis dissimilarity, *P* < 0.05; Fig. 5a). Near-coral seawater taxonomic microbiomes were significantly structured by zone and coral species but not disease state (PERMANOVA; Fig. 5b).

We next identified specific microbial taxa that may be related to the biogeographic environment. We conducted a differential abundance test by zone and identified 12 ASVs that significantly differed in apparently healthy colonies (false discovery rate [FDR] adjusted *P* < 0.05; Fig. 5c). The ASVs that differed included two Clostridiaceae bacteria, which varied in abundance by zone. Several other ASVs also had variable abundances relative to zone 3 (Upper Keys, chosen as the reference zone because it had the highest ratio of heterotrophs: autotrophs and highest organic carbon), including *Alteromonas*, *Paramoritella*, Rickettsiales S25–593, *Balneola*, and *Acinetobacter.* The Pirellulaceae family and SAR11 clade III microbes were depleted at all zones relative to zone 3, while *Rubritalea*, Desulfocapsaceae, and Sandaracinaceae were enriched in all zones relative to zone 3 (Fig. 5c).

## Discussion

We present a comprehensive omic analysis of microorganisms and dissolved metabolites spanning hundreds of kilometers of FCR, which has suffered over the past 50 years from numerous natural and anthropogenic stressors. Following comparative analyses of these omic data sets, we demonstrate that reef water metabolites and microorganisms as well as coral-associated microbes distinguish between biogeographically distributed reef habitats. Further, taxonomic microbiomes and targeted metabolomic methods were related to underlying benthic organisms and environmental features such as organic carbon and abundances of planktonic microbes. These data provide novel insights into reef ecosystem parameters and may be useful for identifying nonvisible changes in coral reef ecosystems.

The biogeographic zone emerged as strongly associated to microbiome and metabolome composition from individual reefs in Florida. These differential patterns across zones, as well as emergent habitat and environmental features, are summarized in Fig. S10. For example, omic signatures in zones 1 and 8 were often opposing and distinct. Indeed, Dry Tortugas National Park (zone 8) emerged as a unique habitat, containing low levels of MTA, a distinct microbial community with low abundances of heterotrophic microbes, and high numbers of functional and taxonomic microbial indicators, including *Synechococcus* and *Alcanivorax*, compared to zone 1, North Key Largo and Biscayne Bay (Fig. S10). Within the Upper to Lower Keys and Marquesas, seawater signatures were likewise distinct, and these zonal signatures were even captured within apparently healthy coral host-associated microbial taxa that shifted across reef zones.

These zonal changes agree with the established importance of biogeography for microorganisms in reef seawater (26, 27, 30). While microbiome studies abound, understanding of biogeographic signatures of reef metabolites is still in its infancy. Similar to our findings, Yamashita et al. (56) found significant shifts in optical properties of seawater dissolved organic matter (DOM) across regions in FCR, largely attributed to differences in anthropogenic and terrestrial influences. In contrast, a study of the high-quality reefs in the Cuban Jardines de la Reina archipelago using the same mass spectrometry-based methods as the present paper did not find a strong biogeographic signature (22). These differences could be related to variation in anthropogenic influence, hydrogeography, and overall quality between the FCR and Jardines de la Reina reef systems (31). As reef metabolomics (both targeted and untargeted approaches) begin to gain traction in reef ecology (18), we anticipate future analyses of dissolved reef metabolites will provide insight into the mechanisms and consequences of such metabolomic and microbial biogeography.

Reef water environmental features of FCR habitats, which changed zonally, explained a larger portion of the variation in microbiomes and metabolomes than other parameters like benthic organisms. The abundance of heterotrophic microbes and the degree to which they outnumbered their photosynthetic counterparts (*Prochlorococcus* and *Synechococcus*) significantly increased northeast along FCR, suggesting the northern region may be more heterotrophic, or the types of autotrophs could be shifting to different phytoplankton groups. Additionally, high TOC concentrations and heterotrophic microbial abundances have been previously documented on Floridian reefs (31), and the pattern of higher TOC in zones 1 and 2 in the Upper Keys and North Key Largo/Biscayne Bay area compared to Dry Tortugas reflects patterns found from years of water quality monitoring in the region (Fig. S11) (38). The increased heterotrophic microbes and TOC in that region explained metabolomic and microbiome compositions of zone 1 (Fig. 1) and was further validated by the five indicator microbes for North Key Largo and Biscayne Bay, which are all known heterotrophs (SAR116, SAR11, NS5 marine group, and Parvibaculales) (Fig. 5). Interestingly, SAR11 clade 1a ASVs were indicators for zone 1, while clades Ib and II were indicative of zone 7, likely reflecting physiological adaptations to the different oceanographic influences of the two regions, or potentially the differing anthropogenic influences between the regions (57). At the western end of FCR, at Dry Tortugas National Park (zone 8), concurrence in the microbial patterns emerged, where *Synechococcus* cells were most abundant and related to microbiome composition. Strain CC9902 was also a microbial indicator for that zone. Fundamental differences in coral growth (40) and nutrient (inorganic and organic) regimes exist between Dry Tortugas, a more offshore environment, and the nearshore North Key Largo/Biscayne Bay area (38), which supports the stark microbial community differences between these habitats.

TOC significantly changed across FCR, but TOC is a single value that does not capture the complex dynamics of thousands of low-molecular-weight dissolved and particulate compounds. After using both targeted and untargeted approaches, we found that dissolved targeted metabolites related well to the benthic and biogeochemical habitats, while the dissolved untargeted metabolome (in both positive and negative ion modes) significantly related to a few measured biogeochemical parameters. Within the untargeted metabolome (positive and negative ion modes), 56 total metabolites shifted across reef zones, in patterns that varied broadly from the concentration of TOC. Two metabolites from the targeted metabolomic analysis, taurocholic acid and MTA, changed significantly across the zones in FCR. Taurocholic acid is a bile salt, which is released and used as an olfactory cue by fishes, and the varied concentrations may reflect differences in fish communities (58), though we did not measure fish biomass or diversity in this study. MTA, a sulfur-containing metabolite, is more concentrated in water overlying reefs compared to deeper, off-reef waters (22) and is cycled by reef sponges (59). Concentrations of MTA in the Marquesas and the Dry Tortugas National Park were similar to the off-reef water measured by Weber et al. (22), perhaps due to the similar open-ocean hydrodynamic regimes. MTA is cycled by several pathways intracellularly, and microbial proteins involved in these pathways were encoded for the seawater metagenomes, including proteins that produce MTA (polyamine aminopropyltransferase, COG0421, EC 2.5.1.16 and EC 2.5.1.104; isovaleryl–homoserine lactone synthase, COG3916, EC 2.3.1.228) and proteins that use MTA (MTA phosphorylase, COG0005, EC 2.4.2.28; MTA/SAM nucleosidase, COG0775, EC 3.2.2.9; MTA/SAM deaminase, COG0402, EC 3.5.4.28). In the present study, the negative relationship between decreasing MTA concentrations and increasing abundances of genes for proteins that use MTA followed an expected pattern, but the opposite correlation was true for proteins that produce MTA (Fig. S9). While we measured extracellular MTA concentrations, our genetic data did not identify any removal pathways from cells (i.e. transporters) and instead identified intracellular proteins found in many bacteria and archaea for recycling MTA so that it does not accumulate in cells and inhibit other cellular pathways (60, 61). Ultimately, given the presence of MTA and MTA cycling genes in reef seawater metagenomes and the known ecological relevance of taurocholic acid, further experimental research on these and other metabolites is warranted to demonstrate the role these metabolites play in reef habitats. With this information, we will be better equipped to disentangle the implications of these metabolite patterns on reef quality.

FCR has been impacted by a years-long highly contagious SCTLD outbreak (41, 62). After sampling apparently healthy and diseased tissue across five zones and five coral species, surprisingly, reef zone was a stronger driver of coral microbiome structure than species and disease, which are known to impact coral microbiomes (46, 63). This zonal influence mirrored changes we also identified in reef water microbiomes and metabolomes (Fig. 1). Geography is increasingly important to coral microbiome signatures, showing temporal stability (64) that extends for years in aquaria-raised corals (65). Within the overall changes, we identified 12 microorganisms from multiple species of apparently healthy corals that shifted across reef zones regardless of species. Many of these microbes frequently associate with corals, including *Paramoritella* (66), *Acinetobacter* (67, 68), *Alteromonas* (69), *Balneola* (70), and *Rubritalea* (71). Given the strong influence of zones on host microbiomes, further studies on microbiomes associated with coral diseases should consider and control for regional changes in microbial communities.

In addition to zone and microbial parameters, both the taxonomic microbiome and targeted metabolome were related to the benthic environment, with both related to hard and soft coral cover. While omic data were driven by zone, benthic community cover was not (Figs. S1 and S4), which may indicate the benthic habitat influences omic parameters in ways distinct from biogeographic signatures. These patterns are likely due to the known impact of coral-derived metabolites on reef water microbial communities (20, 23, 25). An additional interesting relationship emerged between hard coral species richness and the taxonomic microbiome, which likely reflected the heightened hard coral species richness and distinct coral community composition at Dry Tortugas, a region known to promote coral growth that historically had higher hard coral cover (40, 53). Another component of the benthic habitat that was related to omic data was coral disease (SCTLD) prevalence, which significantly changed across zones and peaked in Zone 5, potentially contributing to the biogeographic signatures we documented in reef water microbiomes and metabolomes. For example, one ASV indicator in zone 5 was Cryomorphaceae, a microbe that has been associated with SCTLD disease lesions in previous studies (47, 72). As coral reefs continue to degrade in part due to infectious diseases, implementing omics in reef water in addition to coral evaluations may help capture reef ecosystem changes. While some programs already monitor specific groups of microorganisms of public health interest (73), expanding to multiple omic strategies that could capture microorganisms implicated in marine diseases may shed light into potential mechanisms leading to the reef's condition.

**Fig. 6.**
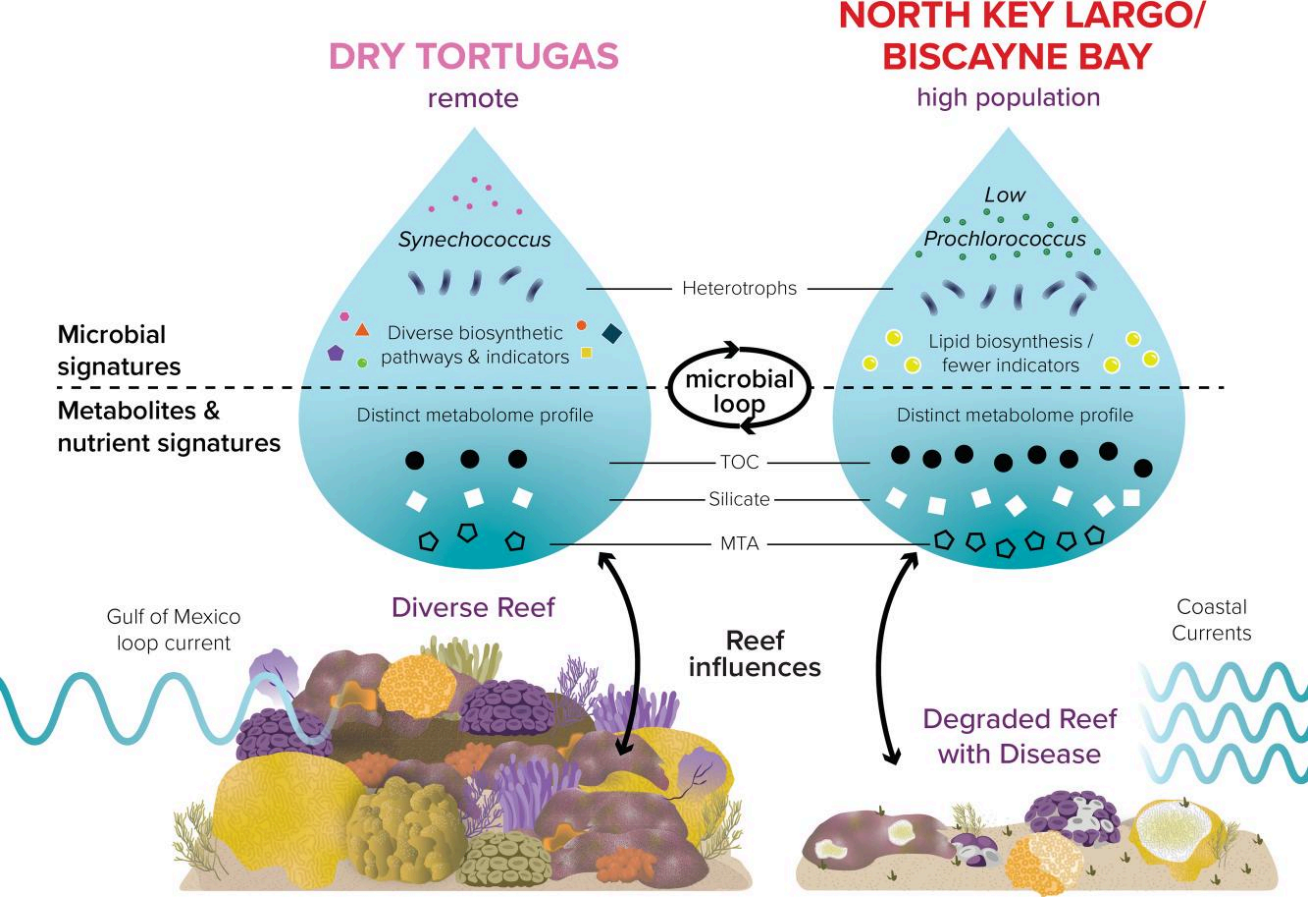
Conceptual diagram of how differential seawater omic signatures depict ocean environment and ecosystem conditions in two contrasting Florida's Coral Reef habitats, Dry Tortugas (zone 8) and North Key Largo/Biscayne Bay (zone 1). Metabolites and microorganisms that are measured by omics and other approaches are connected via the microbial loop. Within two different habitats, the omic signatures become unique as they are influenced by reef habitat and likely by parameters not examined here, such as prevailing water currents and human population density. While habitat conditions influenced seawater omics, next steps include experimental investigations into the mechanisms related to these signatures and determining if these can serve as diagnostic tools for reefs.

While the benthic community changed minimally over the eight zones we studied, the microorganisms, metabolites, and organic nutrients we measured within the overlying water column differed across the zones. For example, at one end of FCR, Dry Tortugas National Park, the waters harbored increased *Synechococcus* and lowered heterotrophic microbes and more diverse microbial taxonomic and biosynthetic pathways indicative of the region (Fig. 6). At the northeastern end of our study area, North Key Largo and Biscayne Bay harbored low abundances of *Prochlorococcus* and increased heterotrophic microorganisms, lipid biosynthesis, and less diverse microbial indicator taxa (Fig. 6). Metabolite and nutrient signatures were also distinct between the two regions (Fig. 6). These seawater-bound features are connected through benthic–pelagic coupling with the underlying reef habitat which varies across reef regions and are likely influenced by anthropogenic impacts like development and population density (26, 70), though that was not measured here. One of the major differences in the benthic habitat was disease history, which may have contributed to these signatures in the reef water. North Key Largo/Biscayne Bay is near the initial outbreak area of SCTLD in 2014 (62) and has therefore experienced multiple years of disease pressure, while Dry Tortugas had no SCTLD. Dry Tortugas corals contracted SCTLD in 2021 (74), and thus our data set captured the multi omic profile of this region ahead of the disease front.

Metabolome and microbiome analyses have the potential to revolutionize our understanding of reef processes and energetics, but our understanding of the current data and their implications is limited, and their inclusion into monitoring programs and research studies comes with significant challenges. Metabolomics alone requires specialized equipment and expertise from sampling to mass spectrometric analysis, and access to and use of such mass spectrometry facilities can be costly and analytically and computationally challenging. To begin, conducting experiments with targeted compounds, like MTA and taurocholic acid, could offer an initial glimpse into how they are implicated in reef processes. Targeted metabolomics utilizes authentic standards to quantify each metabolite and is adaptable and comparable between mass spectrometry facilities. Microbiome analyses are even more developed and accessible, with more facilities and at a lower cost than metabolomics. Characterizing taxonomic microbiomes (via 16S rRNA gene sequencing) is more cost-effective compared to shotgun metagenomic sequencing and is even adaptable to in-the-field use, though at the loss of potentially useful functional information (46, 75). Regardless, given the significant associations and indicators of the taxonomic microbiome with reef zones, benthic organisms, environmental features in this data set, and the relative ease of use compared to other omic techniques, characterizing them in a time-series context with existing monitoring programs could be highly informative for understanding reef dynamics.

In conclusion, this novel omic data set of reef microorganisms and metabolites enhances our understanding of the chemical and microbial habitat within FCR. Further, it provides insights into specific compounds (e.g. MTA and taurocholic acid) and microbial indicators for focus in follow-up experimental studies to better understand the dynamics of these indicators within the reef habitat. Outside of experimental work, continued investigations of reef microorganisms and metabolites are needed. In particular, those in a time-series context that link microbial and metabolite patterns to habitat characteristics or external stressors will help elucidate the mechanisms behind patterns we observed and how these features may contribute to reef decline or growth. For example, currently (summer 2023) FCR is experiencing unprecedented levels of seawater warming and coral bleaching. Follow-up omic studies could identify the nonvisible, ecological impacts of the predicted coral reef ecosystem mortality. While metabolomics and microbial analyses probe the smallest components of a reef, they also reflect larger habitat patterns in reef environments, warranting further inclusion of these techniques in studying Earth's most biodiverse ocean ecosystem.

## Materials and methods

### Study area and sampling

We sampled coral reef benthic environments during a research cruise aboard the M/V *Alucia* between 2019 June 5 and 2019 June 19 (Fig. 1; File S03). We targeted 85 reefs that were separated into eight zones (between 8 and 14 reefs per zone), based on historical management areas that line up with subregions of Biscayne National Park and North Key Largo area; Upper, Middle, and Lower Keys; the Marquesas; and Dry Tortugas National Park, similar to those reported in publications and used in the Disturbance Response Monitoring program (52, 53, 76) (see Methods S02). At 85 reefs, 30-min roving diver surveys were used to measure the prevalence of SCTLD. As of June 2019, none of the colonies surveyed had received any treatment for SCTLD. Within a 100 m^2^ plot, high-resolution imagery for benthic composition was captured on 45 reefs. We collected 30–40 mL of seawater via SCUBA at all 85 reefs at reef depth for inorganic nutrients and organic carbon and total nitrogen (TN). Inorganic nutrient samples were frozen after 1.4 mL subsamples were removed and fixed with paraformaldehyde (1% final concentration) to quantify cell abundances. Organic carbon and TN samples were acidified and kept at room temperature or 4°C until analysis.

At 13 reefs, we collected triplicate 1.7 L seawater samples for metabolomic analysis (targeted and untargeted) via SCUBA. The seawater was prefiltered (0.1 µm) to remove microbial biomass and acidified before low-molecular-weight organic metabolites were concentrated using solid-phase extraction (SPE) cartridges. At 27 reefs (including the 13 sampled for metabolomics), we collected two 4 L benthic seawater samples. We filtered 2 L at a time using a 0.2 µm filter (microbiome analysis) or GF/F filter (chlorophyll analysis). Filters and SPE cartridges were frozen at −80°C for analysis at the Woods Hole Oceanographic Institution (WHOI).

On 11 reefs with active SCTLD, we collected coral tissue with 10-mL Luer Slip syringes and near-coral seawater with 60-mL Luer Lok syringes from separate apparently healthy and SCTLD-afflicted coral colonies. We targeted the following coral species: *Colpophyllia natans*, *Dichocoenia stokesii*, *Montastraea cavernosa*, *Orbicella faveolata*, and *Pseudodiploria strigosa.* The seawater was hand-filtered (0.2 µm), and all samples (filters and coral tissue) were frozen at −80°C until analysis at WHOI. Further details are included in the Methods S02.

### Benthic and seawater environmental sample processing

Photomosaics at 45 reefs were analyzed for benthic composition as described in Fox et al. (77). The number of diseased and apparently healthy coral colonies were counted and used to generate a proportion of colonies at each reef with SCTLD. Samples collected for quantifying microbial cell abundances were processed at the University of Hawaii, as described previously (78). Nonpigmented prokaryotes were used as a proxy for heterotrophic bacterial and archaeal cells (79, 80), referred to as “heterotrophic microbes”. Nonpurgeable TOC, TN, and inorganic nutrients (phosphate, ammonium, silicate, nitrite, and nitrate) were analyzed as described previously (31). TON was calculated by subtracting ammonium and nitrite plus nitrate from TN. Chlorophyll analysis involved a 24-h 90% acetone extraction, followed by fluorometric analysis. See Methods S02 for further details.

### Metabolomic and microbiome laboratory processing

SPE cartridges were processed following the methods in Weber et al. (22). For untargeted metabolite analysis, eluted and resuspended extracts were measured using an ultrahigh-performance liquid chromatography system (Vanquish UHPLC) coupled with an Orbitrap Fusion Lumos Tribrid mass spectrometer that was run in both positive and negative ion modes (see Methods S02). Processing the untargeted data using XCMS (Methods S02) resulted in a final chemical feature table with unique mass-to-charge ratios and retention times for each feature as well as the corresponding peak intensity for that feature across samples. To prepare the untargeted metabolomic data for statistical analyses, data were filtered of blank-associated features and features with low coefficient of variation across samples and then normalized to seawater volume, keeping 1,428 (negative ion mode) and 2,759 (positive ion mode) features. Extracts for targeted metabolomics were analyzed using UHPLC (Accela Open Autosampler and Accela 1250 Pump) coupled with a H-ESI and a triple stage quadrupole mass spectrometer (TSQ Vantage) that was operated in selective reaction monitoring mode. Targeted compounds included a range of environmentally relevant vitamins, amino acids, and other metabolites that have been detected in marine microorganisms, their culture media, and reef seawater (59, 81, 82). After conversion of instrument files, final concentrations were corrected for limit of detection and extraction efficiency (83). See Methods S02 for further details.

We extracted DNA from 0.2 µm filters used for 1.7 L seawater collections and four control blank filters using PowerBiofilm kits (Qiagen, Germantown, MD, USA) following manufacturer protocols. The resulting DNA was used as the template for both 16S rRNA gene sequencing of bacteria and archaea as well as shotgun sequencing. For 16S rRNA gene sequencing for the taxonomic microbiome, we followed methods previously reported (78) and sequenced 2 × 250 bp on an Illumina MiSeq. A library for shotgun metagenomics (functional microbiome) was prepared using the Illumina DNA Prep following manufacturer protocols. Samples were sequenced 2 × 150 bp on a P3 flow cell on an Illumina NextSeq 2000. See Methods S02 for library preparation details.

To expedite the turnaround time between sample collection and taxonomic microbiome results for disease diagnostics, we performed on-ship processing and 2 × 150 bp sequencing on the Illumina iSeq100 system for near-coral seawater samples from five reefs from 2019 June 9 to 2019 June 10. All remaining coral and near-coral seawater samples were processed at WHOI. On-ship and at-WHOI methods followed in-the-field microbiome preparation protocols described previously (46) and are detailed further in the Methods S02.

### Bioinformatics

Sequence reads from the Illumina MiSeq and iSeq runs were separately run through the DADA2 pipeline to generate ASVs (v1.18.0) (84). We removed mitochondrial and chloroplast ASVs and filtered out low-abundant ASVs (average count <0.5). DNA extraction and PCR controls were removed for data analysis. ASVs were given a unique number, and sequences associated with the IDs were saved.

Shotgun sequencing on the Illumina NextSeq yielded 13,592,782 ± 3,357,010 paired-end sequence reads per reef seawater microbial community sample. Due to sequencing errors, six reefs were singulars, while all others had duplicate sequence samples. We quality filtered the sequence reads and coassembled them with MegaHit (v1.2.9) (85), as this assembler has been shown to generate more genes that could be successfully annotated in complex environments such as ocean and soil samples compared to metaSPAdes (86). We annotated the assembly and calculated gene abundances. We kept only genes with known Clusters of Orthologous Genes (COG) identifiers and further filtered to remove low-abundance genes, leaving 103,665 genes. All resultant gene and taxa data tables were transformed to relative abundance and log transformed after adding a pseudocount of 1. See Methods S02 for detailed bioinformatic packages, parameters, and links to scripts.

### Statistical analysis

Benthic cover, hard coral richness, organic and inorganic nutrients, cell abundances, and disease prevalence data were measured from 45 reefs (benthic cover) or 85 reefs (all other parameters) in FCR across eight zones. We used a Kruskal–Wallis test in R (v4.0.3) to compare changes across zones with a Bonferroni-corrected *P*-value to account for multiple comparisons (Fig. 2). We followed with a Wilcoxon rank sum test for pairwise comparisons (significant at *P* < 0.05 after a Benjamini–Hochberg FDR adjustment) (File S05). To examine the changes in benthic composition and hard coral species composition across FCR, we conducted a principal component analysis, followed by a PERMANOVA test by zone. Wilcoxon rank sum tests were used to compare environmental variables at Dry Tortugas National Park to all other zones. Additional linear regressions were conducted between environmental variables (see Methods S02).

A dbRDA was calculated to evaluate how geography (zone) and environmental conditions (TOC, heterotrophic microbes, *Prochlorococcus*, *Synechococcus*, picoeukaryotes, hard coral cover, algae cover, soft coral cover, sponge cover, and hard coral species richness) explained changes in metabolomes and microbiomes. dbRDAs were calculated for each data set with either gower (targeted metabolomes) or Bray–Curtis (other omic data sets) dissimilarities. Significance of individual environmental variables within the analysis was evaluated with an ANOVA using the parameter by = “terms”.

To test for metabolites that changed across zones, sites in zones 2 and 3 as well as zones 5 and 6 were grouped to ensure each zone had more than one reef. Kruskal–Wallis or ANOVA tests were used to evaluate significantly changing metabolites across zones (Bonferroni-corrected *P<* 7.246 × 10^−5^ and 3.378 × 10^−5^ for negative and positive metabolites, respectively), followed by pairwise Wilcoxon rank sum tests between zones (FDR-adjusted *P* < 0.05). The 56 untargeted features were *z*-score standardized for visualization. Model II ordinary least squares linear regressions were used to relate average relative abundance of MTA source and sink genes in seawater metagenomes to dissolved concentration of MTA.

To identify seawater taxa and functional microbiome indicators of FCR, we used the multilevel pattern analysis, multipatt() function in the R package indicspecies (v1.7.12). Indicators were reported if they had a positive predictor value (*A*) over 0.6 and sensitivity (*B*) over 0.6 and had a *P* < 0.05. Indicator ASVs and functional genes were visualized with heat maps and summarized in File S04.

Coral and near-coral seawater taxonomic microbiome beta diversity was calculated with Bray–Curtis dissimilarity and visualized with principal coordinates analysis. A PERMANOVA test was used to identify the influence of zone, disease, and coral species on microbiome composition. Apparently healthy coral microbiomes were subset from the larger data set for differential abundance analysis to test which ASVs significantly changed by zone and controlling for the effect of species using corncob (87). All statistical tests are detailed further in the Methods S02.

## Supplementary Material

pgad287_Supplementary_Data

## Data Availability

Metabolomic data files are uploaded to MetaboLights database and are under curation under accession MTBLS6574. All sequence data (metagenomic and 16S rRNA) are uploaded to the NCBI Sequence Read Archive under BioProject numbers PRJNA910824 and PRJNA742384. Environmental data and metabolite and sequence data accessions can be accessed within BCO-DMO project 746196 under data set 890979. Code for reproducing statistical analyses and main figures in the manuscript are uploaded to https://github.com/CynthiaBecker/FL-reef-omics.

## References

[1] Kittinger JN , et al 2011. Historical reconstruction reveals recovery in hawaiian coral reefs. PLoS One 6:e25460. 21991311 10.1371/journal.pone.0025460 PMC3184997

[2] Gardner TA , Côté IM, Gill JA, Grant A, Watkinson AR. 2003. Long-term region-wide declines in Caribbean corals. Science 301:958–960. 12869698 10.1126/science.1086050

[3] Guest JR , et al 2018. A framework for identifying and characterising coral reef “oases” against a backdrop of degradation. J Appl Ecol. 55:2865–2875.

[4] De’ath G , Fabricius KE, Sweatman H, Puotinen M. 2012. The 27–year decline of coral cover on the Great Barrier Reef and its causes. Proc Natl Acad Sci U S A. 109:17995–17999. 23027961 10.1073/pnas.1208909109 PMC3497744

[5] McClenachan L , O’Connor G, Neal BP, Pandolfi JM, Jackson JBC. 2017. Ghost reefs: nautical charts document large spatial scale of coral reef loss over 240 years. Sci Adv. 3:e1603155. 28913420 10.1126/sciadv.1603155 PMC5587093

[6] Alevizon WS , Porter JW. 2015. Coral loss and fish guild stability on a Caribbean coral reef: 1974–2000. Environ Biol Fish. 98:1035–1045.

[7] Dustan P . 2003. “Ecological perspective: the decline of Carysfort reef, Key Largo, Florida 1975–2000” (NOAA Technical Memorandum NOS NCCOS).

[8] Vermeij MJA , Smith JE, Smith CM, Vega Thurber R, Sandin SA. 2009. Survival and settlement success of coral planulae: independent and synergistic effects of macroalgae and microbes. Oecologia 159:325–336. 19050932 10.1007/s00442-008-1223-7

[9] Lecchini D , Nakamura Y. 2013. Use of chemical cues by coral reef animal larvae for habitat selection. Aquat Biol. 19:231–238.

[10] Sneed JM , Sharp KH, Ritchie KB, Paul VJ. 2014. The chemical cue tetrabromopyrrole from a biofilm bacterium induces settlement of multiple Caribbean corals. Proc Biol Sci. 281:20133086–20133086. 24850918 10.1098/rspb.2013.3086 PMC4046396

[11] Bourne DG , Morrow KM, Webster NS. 2016. Insights into the coral microbiome: underpinning the health and resilience of reef ecosystems. Annu Rev Microbiol. 70:317–340. 27482741 10.1146/annurev-micro-102215-095440

[12] Wada N , et al 2022. High-resolution spatial and genomic characterization of coral-associated microbial aggregates in the coral *Stylophora pistillata*. Sci Adv. 8:eabo2431. 35857470 10.1126/sciadv.abo2431 PMC9258956

[13] Andersson ER , et al 2021. Identifying metabolic alterations associated with coral growth anomalies using 1H NMR metabolomics. Coral Reefs 40:1195–1209.

[14] Sullivan B , Faulkner DJ, Webb L. 1983. Siphonodictidine, a metabolite of the burrowing sponge *Siphonodictyon* sp. that inhibits coral growth. Science 221:1175–1176. 17811521 10.1126/science.221.4616.1175

[15] Pawlik JR , Steindler L, Henkel TP, Beer S, Ilan M. 2007. Chemical warfare on coral reefs: sponge metabolites differentially affect coral symbiosis in situ. Limnol Oceanogr. 52:907–911.

[16] Haas AF , et al 2016. Global microbialization of coral reefs. Nat Microbiol. 1:16042. 27572833 10.1038/nmicrobiol.2016.42

[17] Glasl B , Webster NS, Bourne DG. 2017. Microbial indicators as a diagnostic tool for assessing water quality and climate stress in coral reef ecosystems. Mar Biol. 164:91.

[18] Wegley Kelly L , et al 2021. Molecular commerce on coral reefs: using metabolomics to reveal biochemical exchanges underlying holobiont biology and the ecology of coastal ecosystems. Front Mar Sci. 8:630799.

[19] Wegley Kelly L , et al 2022. Distinguishing the molecular diversity, nutrient content, and energetic potential of exometabolomes produced by macroalgae and reef-building corals. Proc Natl Acad Sci USA. 119:e2110283119. 10.1073/pnas.2110283119 PMC8812564 35101918

[20] Nelson CE , et al 2013. Coral and macroalgal exudates vary in neutral sugar composition and differentially enrich reef bacterioplankton lineages. ISME J. 7:962–979. 23303369 10.1038/ismej.2012.161 PMC3635233

[21] Deutsch JM , et al 2021. Metabolomics of healthy and stony coral tissue loss disease affected *Montastraea cavernosa* corals. Front Mar Sci. 8:714778.

[22] Weber L , et al 2020. Extracellular reef metabolites across the protected Jardines de la Reina, Cuba reef system. Front Mar Sci. 7:582161.

[23] Nakajima R , et al 2018. Release of dissolved and particulate organic matter by the soft coral Lobophytum and subsequent microbial degradation. J Exp Mar Biol Ecol. 504:53–60.

[24] Ochsenkühn MA , Schmitt-Kopplin P, Harir M, Amin SA. 2018. Coral metabolite gradients affect microbial community structures and act as a disease cue. Commun Biol. 1:184. 30417121 10.1038/s42003-018-0189-1 PMC6218554

[25] Weber L , et al 2022. Benthic exometabolites and their ecological significance on threatened Caribbean coral reefs. ISME Commun. 2:101. 10.1038/s43705-022-00184-7 PMC9723752 37938276

[26] Kelly LW , et al 2014. Local genomic adaptation of coral reef-associated microbiomes to gradients of natural variability and anthropogenic stressors. Proc Natl Acad Sci U S A. 111:10227–10232. 24982156 10.1073/pnas.1403319111 PMC4104888

[27] Apprill A , et al 2021. Microbial ecology of coral-dominated reefs in the federated states of Micronesia. Aquat Microb Ecol. 86:115–136.

[28] Glasl B , et al 2019. Microbial indicators of environmental perturbations in coral reef ecosystems. Microbiome 7:94. 31227022 10.1186/s40168-019-0705-7 PMC6588946

[29] Laas P , et al 2021. Composition of prokaryotic and eukaryotic microbial communities in waters around the Florida Reef Tract. Microorganisms 9:1120. 34064293 10.3390/microorganisms9061120 PMC8224282

[30] Ma L , et al 2022. Biogeography of reef water microbes from within-reef to global scales. Aquat Microb Ecol. 88:81–94.

[31] Weber L , et al 2020. Microbial signatures of protected and impacted Northern Caribbean reefs: changes from Cuba to the Florida Keys. Environ Microbiol. 22:499–519. 31743949 10.1111/1462-2920.14870 PMC6972988

[32] Roach TNF , et al 2020. A multiomic analysis of in situ coral–turf algal interactions. Proc Natl Acad Sci U S A. 117:13588–13595. 32482859 10.1073/pnas.1915455117 PMC7306781

[33] Little M , et al 2021. Three-dimensional molecular cartography of the Caribbean reef-building coral *Orbicella faveolata*. Front Mar Sci. 8:627724.

[34] Lee TN , Leaman K, Williams E, Berger T, Atkinson L. 1995. Florida Current meanders and gyre formation in the southern straits of Florida. J Geophys Res. 100:8607.

[35] Fratantoni PS , Lee TN, Podesta GP, Muller-Karger F. 1998. The influence of Loop Current perturbations on the formation and evolution of Tortugas eddies in the southern straits of Florida. J Geophys Res. 103:24759–24779.

[36] Kourafalou VH , Kang H. 2012. Florida Current meandering and evolution of cyclonic eddies along the Florida Keys Reef Tract: are they interconnected?: FLORIDA CURRENT AND CYCLONIC EDDIES. J Geophys Res. 117:5028.

[37] Briceño HO , Boyer JN. 2018. “2017 Annual report of the water quality monitoring project for the water quality protection program of the Florida keys National Marine Sanctuary”.

[38] Briceño HO , Boyer JN, Castro J, Harlem P. 2013. Biogeochemical classiﬁcation of south Florida's estuarine and coastal waters. Mar Pollut Bull. 75:187–204. 23968989 10.1016/j.marpolbul.2013.07.034

[39] Toth LT , Kuffner IB, Stathakopoulos A, Shinn EA. 2018. A 3,000-year lag between the geological and ecological shutdown of Florida's Coral Reef. Glob Change Biol. 24:5471–5483. 10.1111/gcb.14389 30133073

[40] Kuffner IB , Stathakopoulos A, Toth LT, Bartlett LA. 2020. Reestablishing a stepping-stone population of the threatened elkhorn coral *Acropora palmata* to aid regional recovery. Endang Species Res. 43:461–473.

[41] Muller EM , Sartor C, Alcaraz NI, van Woesik R. 2020. Spatial epidemiology of the stony-coral-tissue-loss disease in Florida. Front Mar Sci. 7:163.

[42] Neely KL , Lewis CL, Lunz KS, Kabay L. 2021. Rapid population decline of the pillar coral *Dendrogyra cylindrus* along the Florida Reef Tract. Front Mar Sci. 8:656515.

[43] Ushijima B , et al 2020. Disease diagnostics and potential coinfections by *Vibrio coralliilyticus* during an ongoing coral disease outbreak in Florida. Front Microbiol. 11:569354. 33193161 10.3389/fmicb.2020.569354 PMC7649382

[44] Ushijima B , et al 2023. Chemical and genomic characterization of a potential probiotic treatment for stony coral tissue loss disease. Commun Biol. 6:248. 37024599 10.1038/s42003-023-04590-y PMC10079959

[45] Aeby GS , et al 2019. Pathogenesis of a tissue loss disease affecting multiple species of corals along the Florida Reef Tract. Front Mar Sci. 6:678.

[46] Becker CC , Brandt M, Miller CA, Apprill A. 2022. Microbial bioindicators of stony coral tissue loss disease identified in corals and overlying waters using a rapid field-based sequencing approach. Environ Microbiol. 24:1166–1182. 34431191 10.1111/1462-2920.15718

[47] Meyer JL , et al 2019. Microbial community shifts associated with the ongoing stony coral tissue loss disease outbreak on the Florida Reef Tract. Front Microbiol. 10:2244. 31608047 10.3389/fmicb.2019.02244 PMC6769089

[48] Rosales SM , Clark AS, Huebner LK, Ruzicka RR, Muller EM. 2020. *Rhodobacterales* and *Rhizobiales* are associated with stony coral tissue loss disease and its suspected sources of transmission. Front Microbiol. 11:681. 32425901 10.3389/fmicb.2020.00681 PMC7212369

[49] Rosales SM , et al 2023. A meta-analysis of the stony coral tissue loss disease microbiome finds key bacteria in unaffected and lesion tissue in diseased colonies. ISME Commun. 3:19. 36894742 10.1038/s43705-023-00220-0 PMC9998881

[50] Clark AS , et al 2021. Characterization of the microbiome of corals with stony coral tissue loss disease along Florida's Coral Reef. Microorganisms 9:2181. 34835306 10.3390/microorganisms9112181 PMC8623284

[51] Dennis B , Ajasa A, Mooney C. 2023. As Florida ocean temperatures soar, a race to salvage imperiled corals. *Washington Post*.

[52] Burman S , Aronson R, van Woesik R. 2012. Biotic homogenization of coral assemblages along the Florida Reef Tract. Mar Ecol Prog Ser. 467:89–96.

[53] Murdoch TJT , Aronson RB. 1999. Scale-dependent spatial variability of coral assemblages along the Florida Reef Tract. Coral Reefs 18:341–351.

[54] van Woesik R , Roth LM, Brown EJ, McCaffrey KR, Roth JR. 2020. Niche space of corals along the Florida Reef Tract. PLoS One. 15:e0231104. 32255794 10.1371/journal.pone.0231104 PMC7138326

[55] Callahan BJ , McMurdie PJ, Holmes SP. 2017. Exact sequence variants should replace operational taxonomic units in marker-gene data analysis. ISME J. 11:2639–2643. 28731476 10.1038/ismej.2017.119 PMC5702726

[56] Yamashita Y , Boyer JN, Jaffé R. 2013. Evaluating the distribution of terrestrial dissolved organic matter in a complex coastal ecosystem using fluorescence spectroscopy. Cont Shelf Res. 66:136–144.

[57] Carlson CA , et al 2009. Seasonal dynamics of SAR11 populations in the euphotic and mesopelagic zones of the northwestern Sargasso Sea. ISME J. 3:283–295. 19052630 10.1038/ismej.2008.117

[58] Buchinger TJ , Li W, Johnson NS. 2014. Bile salts as semiochemicals in fish. Chem Senses. 39:647–654. 25151152 10.1093/chemse/bju039

[59] Fiore CL , Freeman CJ, Kujawinski EB. 2017. Sponge exhalent seawater contains a unique chemical profile of dissolved organic matter. PeerJ 5:e2870. 28097070 10.7717/peerj.2870 PMC5234435

[60] Parveen N , Cornell KA. 2011. Methylthioadenosine/S-adenosylhomocysteine nucleosidase, a critical enzyme for bacterial metabolism: involvement of MTA/SAH nucleosidase in bacterial metabolism. Mol Microbiol. 79:7–20. 21166890 10.1111/j.1365-2958.2010.07455.x PMC3057356

[61] Sauter M , Moffatt B, Saechao MC, Hell R, Wirtz M. 2013. Methionine salvage and *S*-adenosylmethionine: essential links between sulfur, ethylene and polyamine biosynthesis. Biochem J. 451:145–154. 23535167 10.1042/BJ20121744

[62] Precht WF , Gintert BE, Robbart ML, Fura R, van Woesik R. 2016. Unprecedented disease-related coral mortality in southeastern Florida. Sci Rep. 6:31374. 27506875 10.1038/srep31374 PMC4979204

[63] Hernandez-Agreda A , Leggat W, Bongaerts P, Herrera C, Ainsworth TD. 2018. Rethinking the coral microbiome: simplicity exists within a diverse microbial biosphere. mBio. 9:e00812-18. 30301849 10.1128/mBio.00812-18 PMC6178627

[64] Epstein HE , et al 2019. Temporal variation in the microbiome of acropora coral species does not reflect seasonality. Front Microbiol. 10:1775. 31474944 10.3389/fmicb.2019.01775 PMC6706759

[65] Williams SD , et al 2022. Geographically driven differences in microbiomes of *Acropora cervicornis* originating from different regions of Florida's Coral Reef. PeerJ 10:e13574. 35729906 10.7717/peerj.13574 PMC9206844

[66] Hosoya S , Suzuki S, Adachi K, Matsuda S, Kasai H. 2009. *Paramoritella alkaliphila* gen. nov., sp. nov., a member of the family Moritellaceae isolated in the Republic of Palau. Int J Syst Evol Microbiol. 59:411–416. 19196787 10.1099/ijs.0.65809-0

[67] Pereira LB , Palermo BRZ, Carlos C, Ottoboni LMM. 2017. Diversity and antimicrobial activity of bacteria isolated from different Brazilian coral species. FEMS Microbiol Lett. 364:16. 10.1093/femsle/fnx164 28873945

[68] Yang S-H , et al 2020. Locality effect of coral-associated bacterial community in the Kuroshio Current from Taiwan to Japan. Front Ecol Evol. 8:11.

[69] Raina J-B , Tapiolas D, Willis BL, Bourne DG. 2009. Coral-associated bacteria and their role in the biogeochemical cycling of Sulfur. Appl Environ Microbiol. 75:3492–3501. 19346350 10.1128/AEM.02567-08 PMC2687302

[70] Ziegler M , et al 2016. Coral microbial community dynamics in response to anthropogenic impacts near a major city in the central Red Sea. Mar Pollut Bull. 105:629–640. 26763316 10.1016/j.marpolbul.2015.12.045

[71] van de Water JAJM , Coppari M, Enrichetti F, Ferrier-Pagès C, Bo M. 2020. Local conditions influence the prokaryotic communities associated with the mesophotic black coral *Antipathella subpinnata*. Front Microbiol. 11:20. 33123099 10.3389/fmicb.2020.537813 PMC7573217

[72] Huntley N , et al 2022. Experimental transmission of stony coral tissue loss disease results in differential microbial responses within coral mucus and tissue. ISME Commun. 2:46. 10.1038/s43705-022-00126-3 PMC9723713 37938315

[73] Seruge J , et al 2019. Application of a rapid qPCR method for enterococci for beach water quality monitoring purposes in Hawaii: loss of DNA during the extraction protocol due to coral sands. Mar Pollut Bull. 149:110631.

[74] Dobbelaere T , et al 2022. Connecting the dots: transmission of stony coral tissue loss disease from the Marquesas to the Dry Tortugas. Front Mar Sci. 9:778938.

[75] Apprill A . 2019. On-site sequencing speeds up and re-directs field-based microbiology. Env Microbiol Rep. 11:45–47. 30689306 10.1111/1758-2229.12732

[76] Stein J . 2023. “Florida reef resilience program disturbance response monitoring quick look report: summer 2019” (NOAA coral reef conservation program, 2019) (July 17, 2023).

[77] Fox MD , et al 2019. Limited coral mortality following acute thermal stress and widespread bleaching on Palmyra Atoll, Central Pacific. Coral Reefs 38:701–712.

[78] Becker C , et al 2020. Microbial and nutrient dynamics in mangrove, reef, and seagrass waters over tidal and diurnal time scales. Aquat Microb Ecol. 85:101–119.

[79] Monger BC , Landry MR. 1993. Flow cytometric analysis of marine bacteria with Hoechst 33342. Appl Environ Microbiol. 59:905–911. 16348898 10.1128/aem.59.3.905-911.1993 PMC202206

[80] Marie D , Partensky F, Jacquet S, Vaulot D. 1997. Enumeration and cell cycle analysis of natural populations of marine picoplankton by flow cytometry using the nucleic acid stain SYBR green I. Appl Environ Microbiol. 63:186–193. 16535483 10.1128/aem.63.1.186-193.1997 PMC1389098

[81] Fiore CL , Longnecker K, Kido Soule MC, Kujawinski EB. 2015. Release of ecologically relevant metabolites by the cyanobacterium *Synechococcus elongatus* CCMP 1631: metabolomics of *Synechococcus*. Environ Microbiol. 17:3949–3963. 25970745 10.1111/1462-2920.12899

[82] Kido Soule MC , Longnecker K, Johnson WM, Kujawinski EB. 2015. Environmental metabolomics: analytical strategies. Mar Chem. 177:374–387.

[83] Johnson WM , Kido Soule MC, Kujawinski EB. 2017. Extraction efficiency and quantification of dissolved metabolites in targeted marine metabolomics. Limnol Oceanogr Methods. 15:417–428.

[84] Callahan BJ , et al 2016. DADA2: high-resolution sample inference from Illumina amplicon data. Nat Methods. 13:581–583. 27214047 10.1038/nmeth.3869 PMC4927377

[85] Li D , Liu C-M, Luo R, Sadakane K, Lam T-W. 2015. MEGAHIT: an ultra-fast single-node solution for large and complex metagenomics assembly via succinct *de Bruijn* graph. Bioinformatics 31:1674–1676. 25609793 10.1093/bioinformatics/btv033

[86] Quince C , Walker AW, Simpson JT, Loman NJ, Segata N. 2017. Shotgun metagenomics, from sampling to analysis. Nat Biotechnol. 35:833–844. 28898207 10.1038/nbt.3935

[87] Martin BD , Witten D, Willis AD. 2020. Modeling microbial abundances and dysbiosis with beta-binomial regression. Ann Appl Stat. 14:94–115. 32983313 10.1214/19-aoas1283 PMC7514055

