# Supplementary material for: Microorganisms and dissolved metabolites distinguish Florida's Coral Reef habitats
Source: PNAS Nexus. 2023 Sep 5;2(9):pgad287. doi: 10.1093/pnasnexus/pgad287 (PMC10504872; doi:10.1093/pnasnexus/pgad287)
Supplement: pgad287_Supplementary_Data [file pgad287_supplementary_data.zip › PNASNEXUS-PNASNEXUS-2023-00274R-s01.docx]

**Supplementary Tables and Figures**

**Microorganisms and dissolved metabolites distinguish Florida’s Coral Reef habitats**

Cynthia C. Becker^1,2^, Laura Weber^1^, Brian Zgliczynski^3^, Chris Sullivan^3^, Stuart Sandin^3^, Erinn Muller^4,5^, Abigail S. Clark^4,6^, Melissa C. Kido Soule^1^, Krista Longnecker^1^, Elizabeth B. Kujawinski^1^, Amy Apprill^1*^

^1^Marine Chemistry & Geochemistry Department, Woods Hole Oceanographic Institution, Woods Hole, MA 02543, USA

^2^MIT-WHOI Joint Program in Oceanography/Applied Ocean Science & Engineering, Cambridge, MA and Woods Hole, MA, USA

^3^Scripps Institution of Oceanography, University of California San Diego, La Jolla, CA 92093, USA

^4^Elizabeth Moore International Center for Coral Reef Research and Restoration, Mote Marine Laboratory, Summerland Key, FL 33042, USA

^5^Mote Marine Laboratory, Sarasota, FL 34236, USA

^6^The College of the Florida Keys, Key West, FL 33040, USA

*Corresponding author Amy Apprill

**Table S1**. PERMANOVA results from untargeted metabolomics analysis.

| Variable | Untargeted metabolomes - positive ion mode | | Untargeted metabolomes -negative ion mode | |
| --- | --- | --- | --- | --- |
| PERMANOVA variable | p-value | R^2^ | p-value | R^2^ |
| Reef | **0.006** | 0.441 | **<0.001** | 0.516 |
| Zone | **0.023** | 0.217 | **<0.001** | 0.277 |


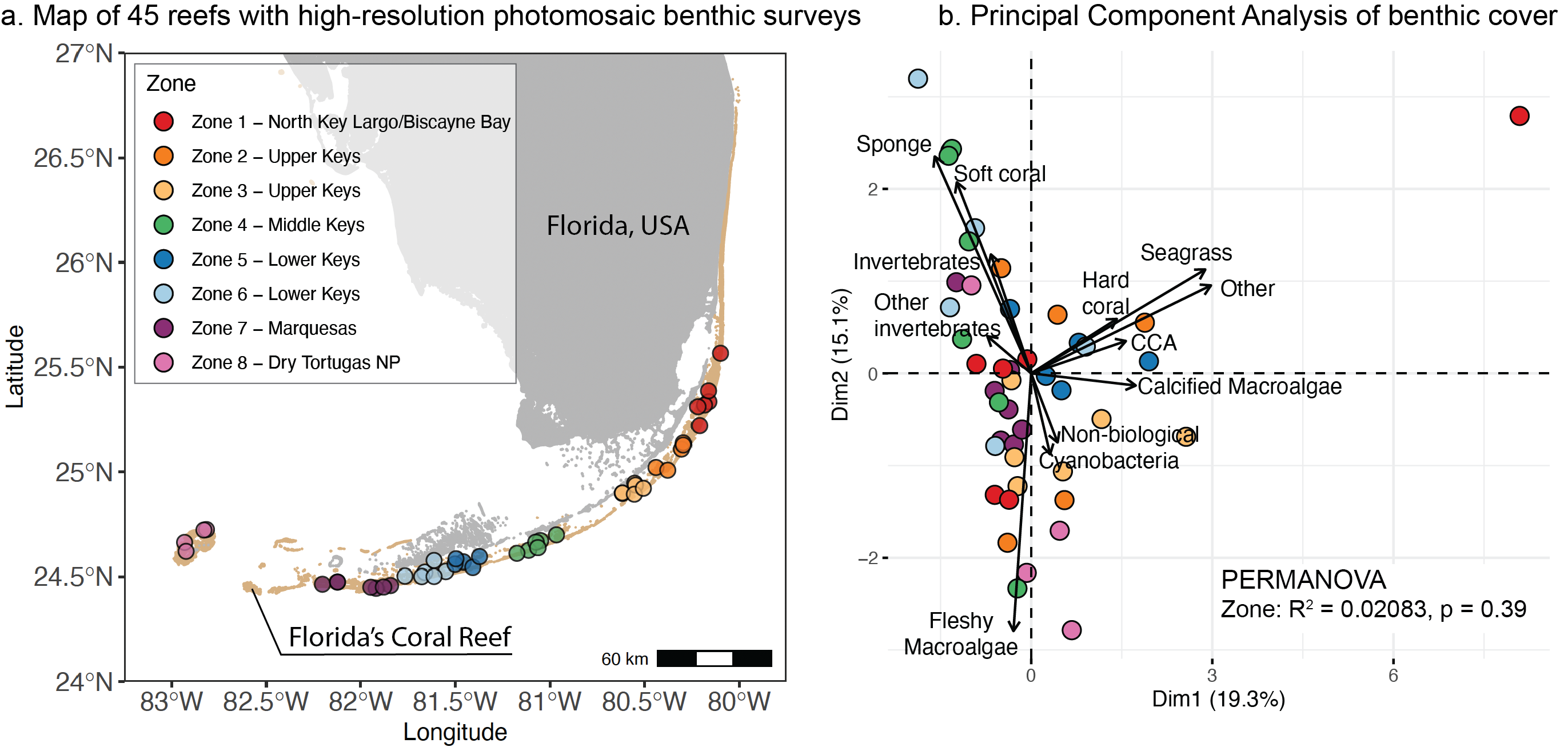


**Figure S1**. a) Reefs (45) sampled for high-resolution photomosaics of 100 m^2^ plots of reef were analyzed for coverage of benthic organisms, which did not change significantly by reef zone as seen by (b) principal components analysis of coral benthic cover (PERMANOVA results of zone on PCA of benthic cover was not significant, p = 0.39).


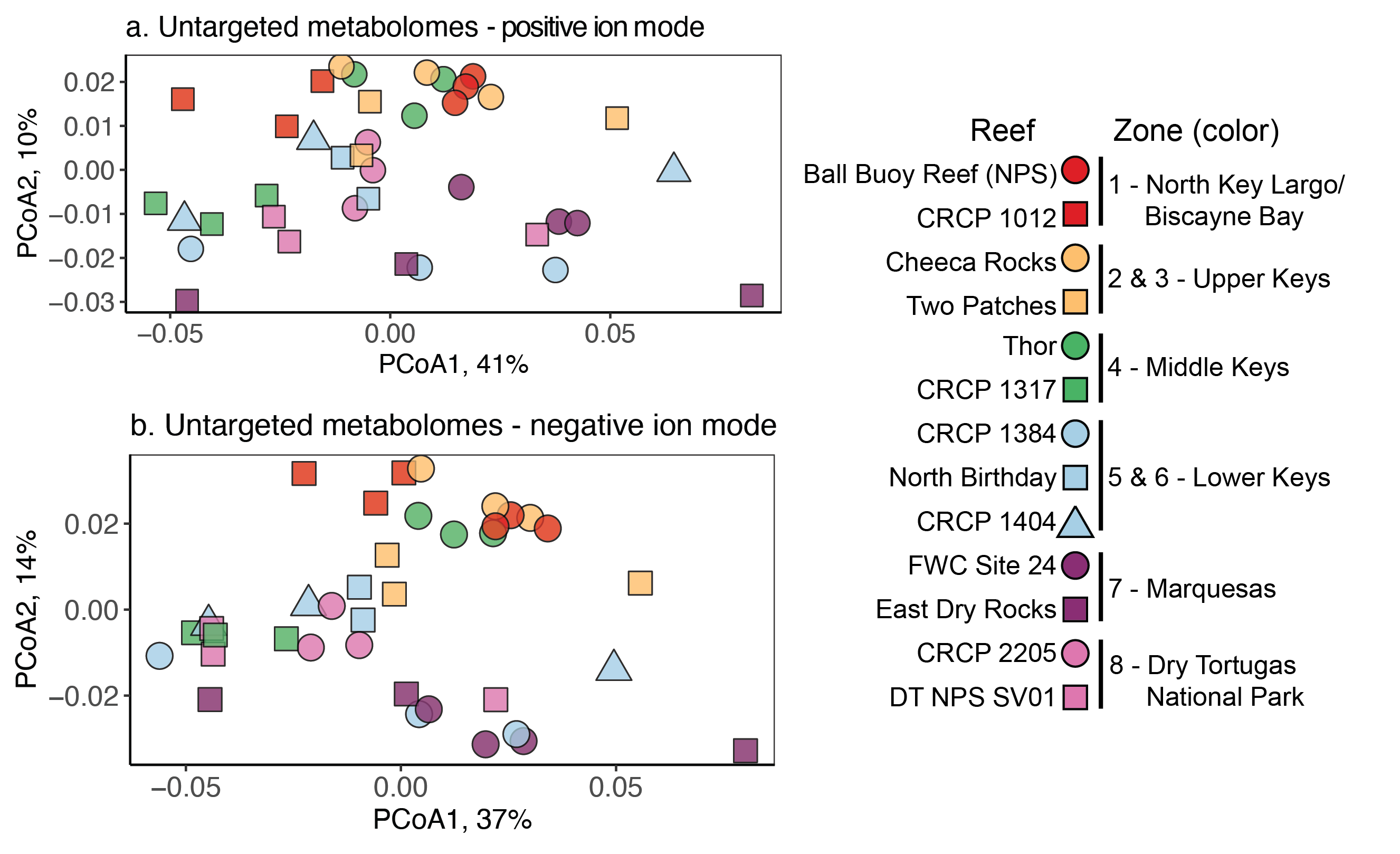


**Figure S2.** Principal coordinates analysis (PCoA) of untargeted metabolite composition for metabolites that ionized in (a) positive ion mode and (b) negative ion mode, displayed according to reef zone (color) and site (symbol). Negative ion mode revealed the most structuring by individual reef, then by reef zone (color) (PERMANOVA p < 0.001 – Table S1).


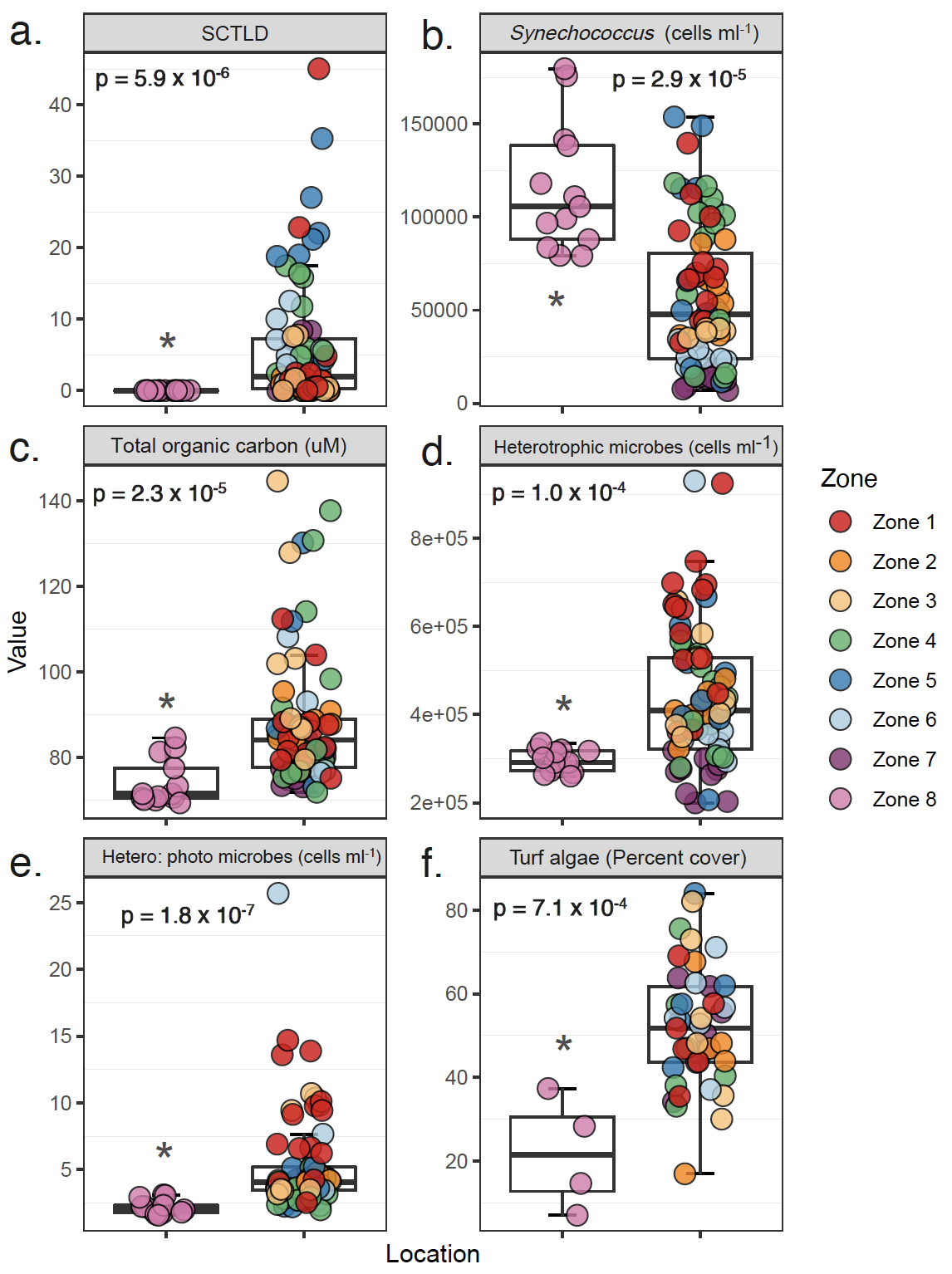


**Figure S3**. Environmental parameters that were significantly different (Wilcoxon Rank Sum test, evaluated at a Bonferroni-correction *p* < 0.001667) at Dry Tortugas National Park (Zone 8, pink) reefs compared to reefs in Zones 1-7 (all other colors). Parameters included (a) stony coral tissue loss disease (SCTLD) prevalence, (b) abundances of *Synechococcus*, (c) concentrations of total organic carbon, (d) abundances of heterotrophic microbes, (e) the ratio of heterotrophic: photosynthetic microbes, and (f) turf algal cover. * = significantly different at Bonferroni-corrected *p* < 0.001667


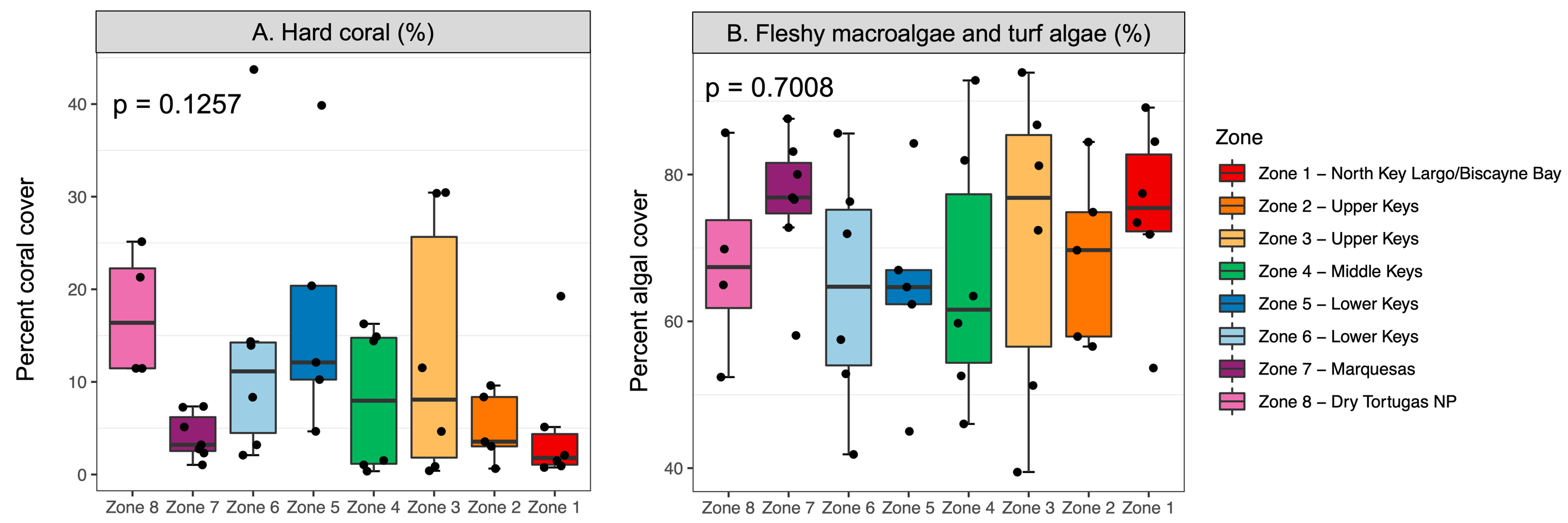
**Figure S4**. Benthic hard coral (a) and algal (b) coverage do not significantly change by zone via Kruskal-Wallis test (Bonferroni-corrected *p* > 0.00192). Benthic cover, given as percent cover, was measured at 45 reefs across 8 different zones represented by different colors (Figure S4a). Box and whisker plots depict the center line, representing the median. Boxes extend from the 1^st^ to 3^rd^ quartiles and the whiskers extend 1.5 x interquartile range. Further analyses of all other individual benthic components (graphs not shown) did not significantly differ by Florida’s Coral Reef zones (Kruskal-Wallis test, Bonferroni-corrected p-values given in each subplot).


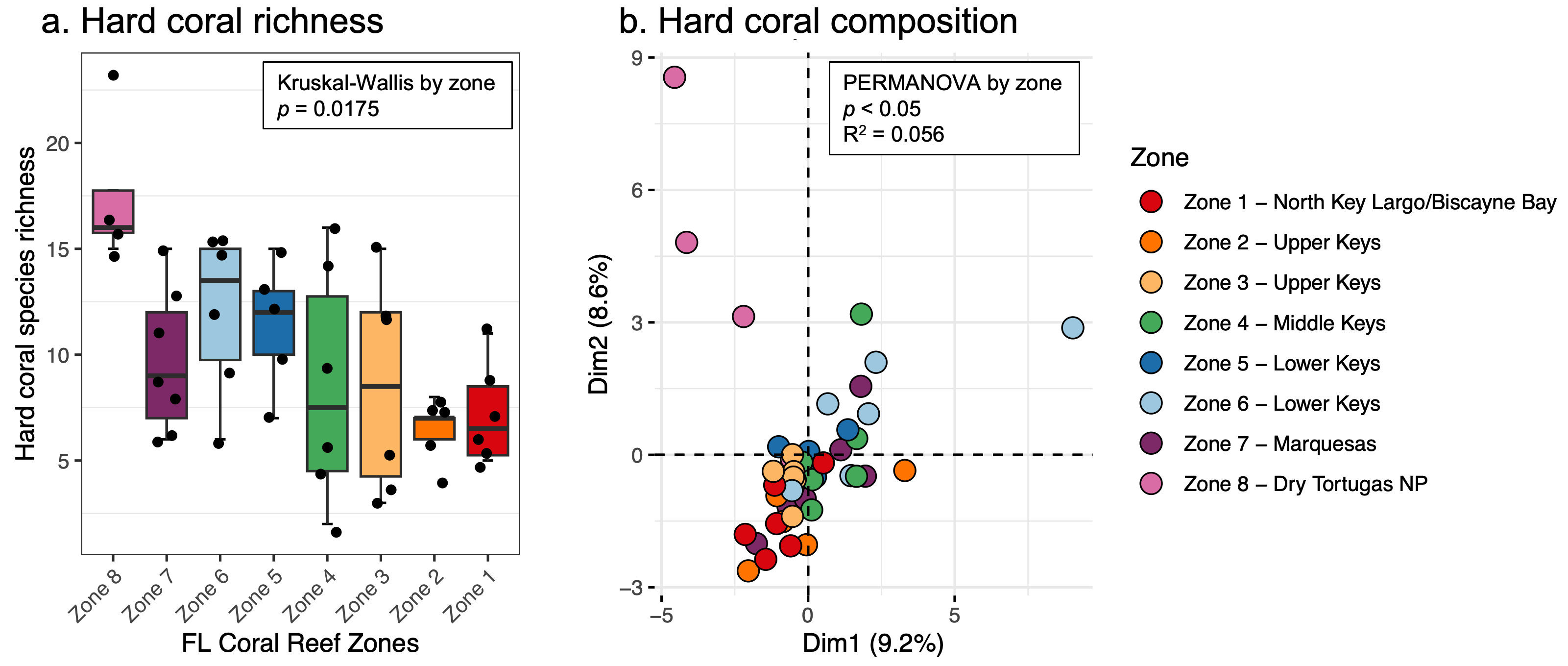


**Figure S5**. Hard coral species richness and composition. Hard coral species were classified down to the lowest taxonomic level and enumerated from a 100 m^2^ plot captured by the high resolution photomosaics (see SI Method). Hard coral species richness (a.) across reefs shifted, but this was not significant at a Bonferroni corrected p-value of 0.00192, which was corrected for multiple comparisons due to the number of environmental variables tested in the overall data collection, and post-hoc pairwise tests yielded no significant relationships (Kruskal-Wallis test followed by Wilcoxon post-hoc test using Benjamini-Hochberg adjusted p-values). Principal components analysis (b.) of hard coral composition revealed significant structuring by zone (PERMANOVA *p* < 0.05, R^2^ = 0.056).


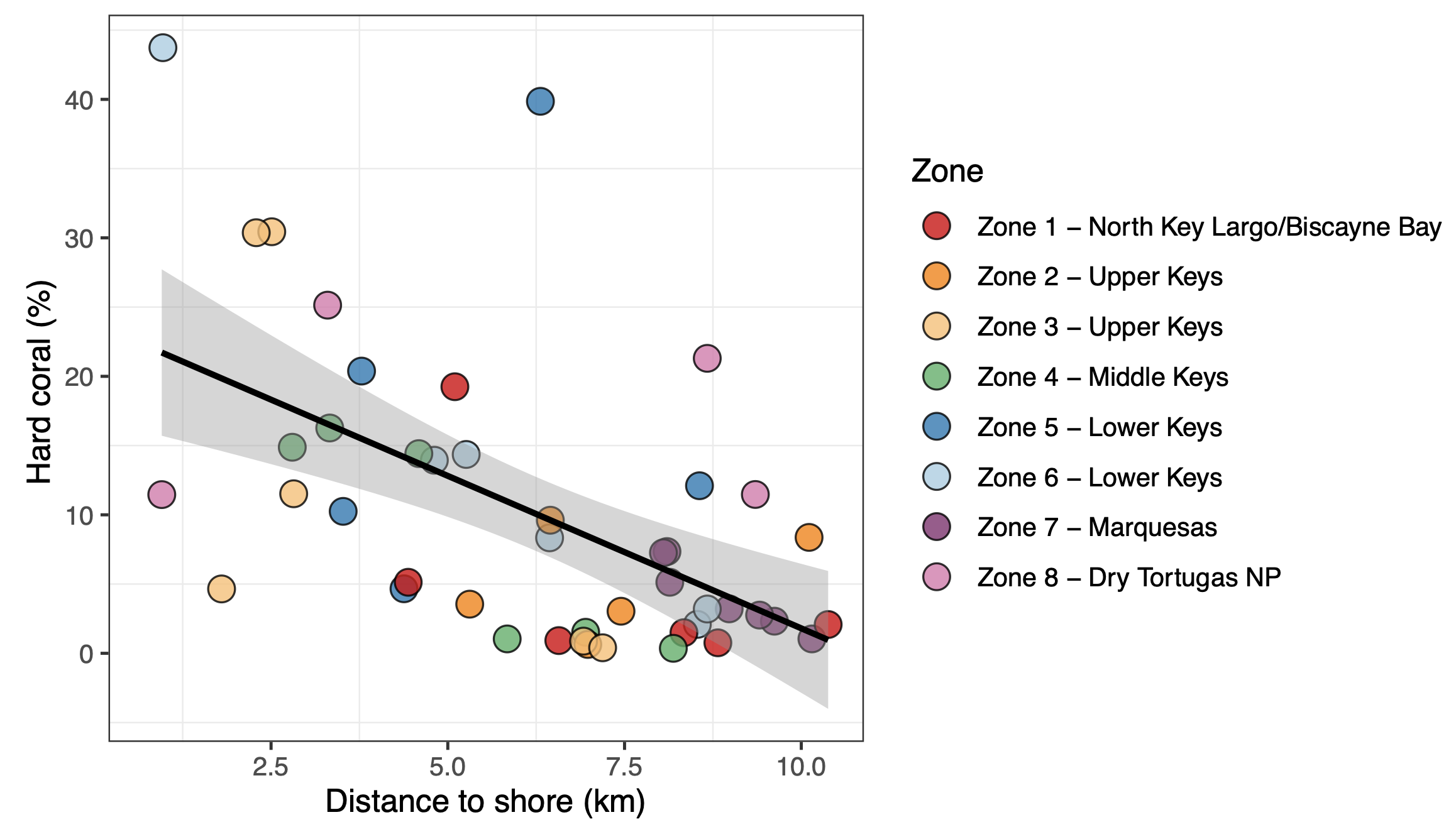


**Figure S6**. Hard coral coverage on Florida’s Coral Reefs significantly decreases as distance from shore increases (linear regression, p < 0.05). Points are colored by zone. Shaded area indicates standard error. Black line is the line of best fit following a linear regression.


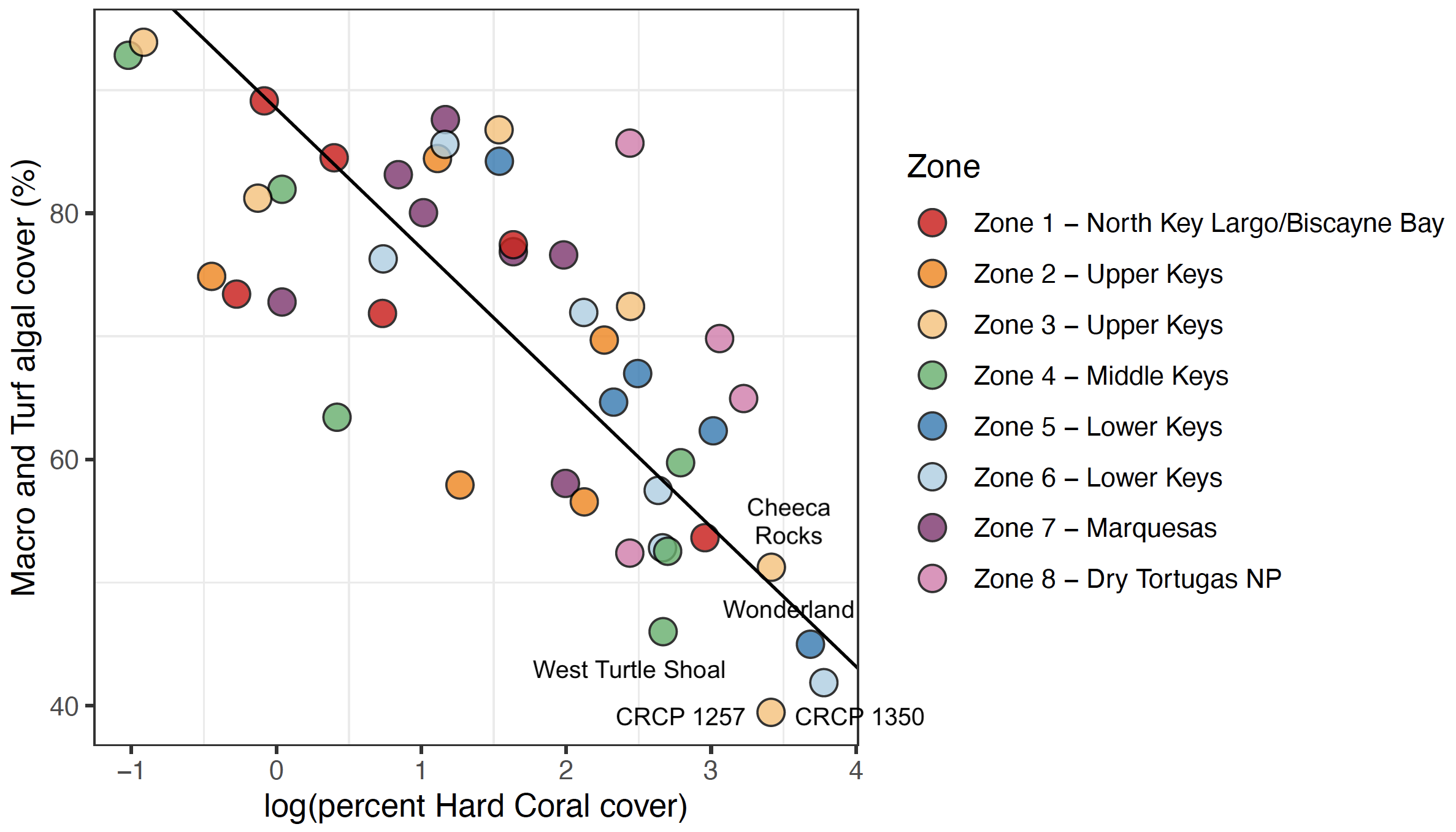


**Figure S7**. Increasing hard coral coverage significantly correlates with decreasing algal coverage (p < 0.001, R^2^ = 0.55). Correlation is with a model II standard major axis regression. The resulting *p* value displayed is from a 1-tailed parametric test using 999 permutations from a major axis model II regression (*p* value cannot be tested on standard major axis regression). Hard coral is presented as the log of percent hard coral cover. Reefs with the combination of highest hard coral cover and lowest algal cover (defined as both fleshy macroalgae and turf algae combined) are labeled.


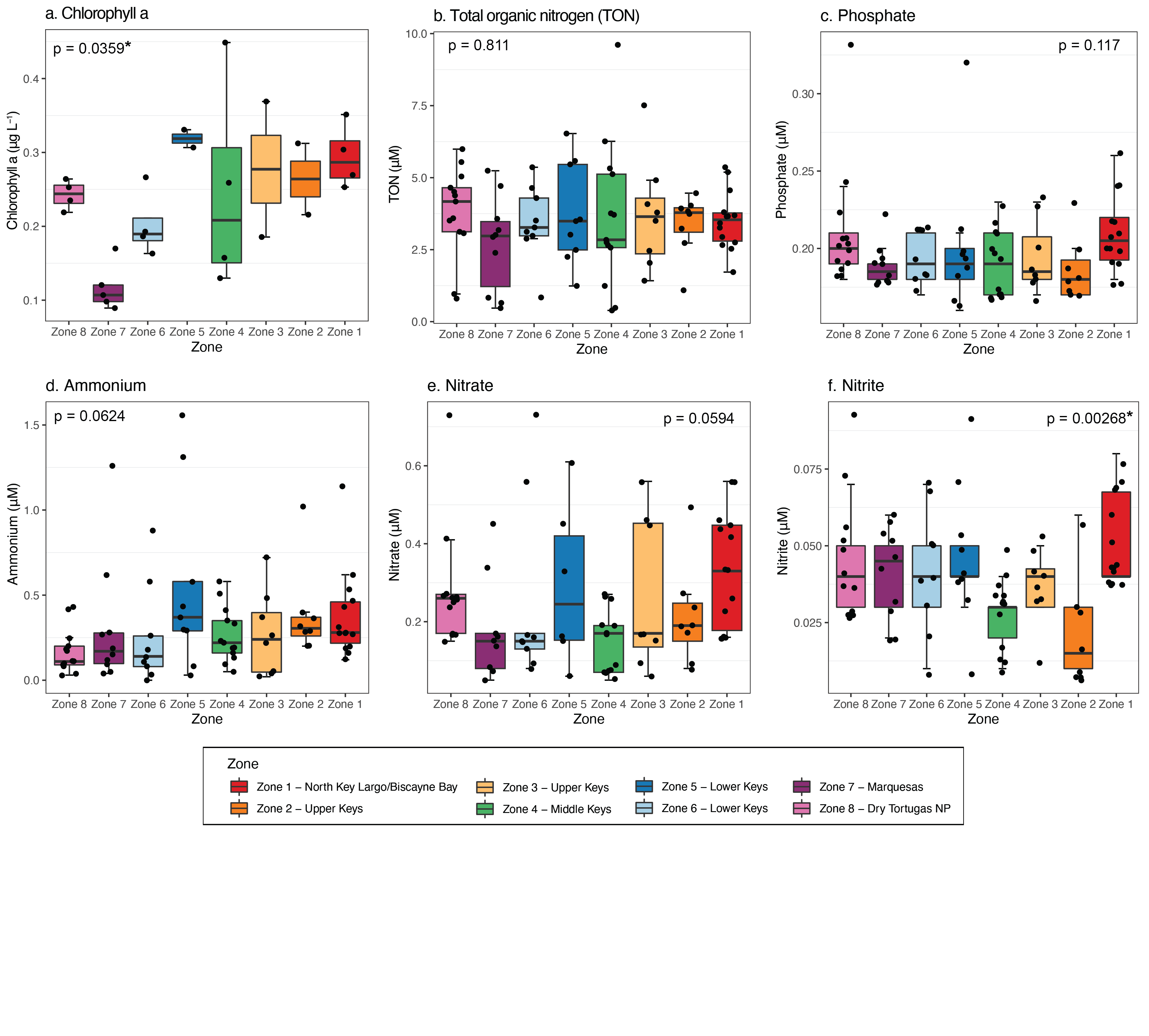


**Figure S8**. Concentration of chlorophyll a and micronutrients across 85 reefs in Florida’s Coral Reef did not change significantly by zone via Kruskal-Wallis test (Bonferroni corrected *p* > 0.00192). Box and whisker plots depict the center line, representing the median. Boxes extend from the 1^st^ to 3^rd^ quartiles and the whiskers extend 1.5 x interquartile range. * = *p* < 0.05, but not smaller than Bonferroni-corrected *p*


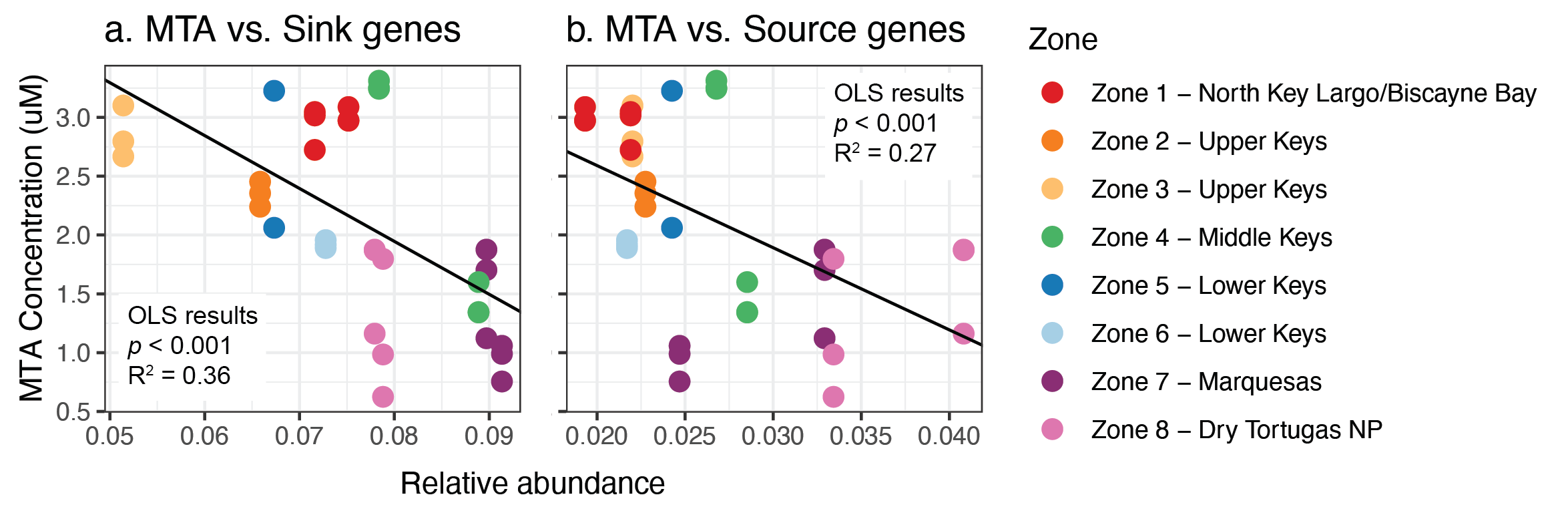


**Figure S9**. Abundance of genes that consume (sink, a) and produce (source, b) MTA are significantly negatively correlated with extracellular concentration of MTA. Correlation is with a model II ordinary least squares (OLS) regression. MTA concentration shown is the square root of the original concentration. Sink genes found in the seawater metagenomes include MTA phosphorylase (COG0005, EC 2.4.2.28), MTA/SAM nucleosidase (COG0775, EC 3.2.2.9), and MTA/SAM deaminase (COG0402, EC 3.5.4.28). Source genes found in the seawater metagenomes include polyamine aminopropyltransferase (COG0421, EC 2.5.1.16, EC 2.5.1.104) and Isovaleryl-homoserine lactone synthase (COG3916, EC 2.3.1.228). MTA = 5'-methylthioadenosine. SAM = S-adenosylhomocysteine. Relative abundances of source and sink genes were averaged between technical replicates for each reef. Concentrations shown are from separate biological triplicates from a reef. The resulting *p* value displayed is from a 1-tailed parametric test using 999 permutations.


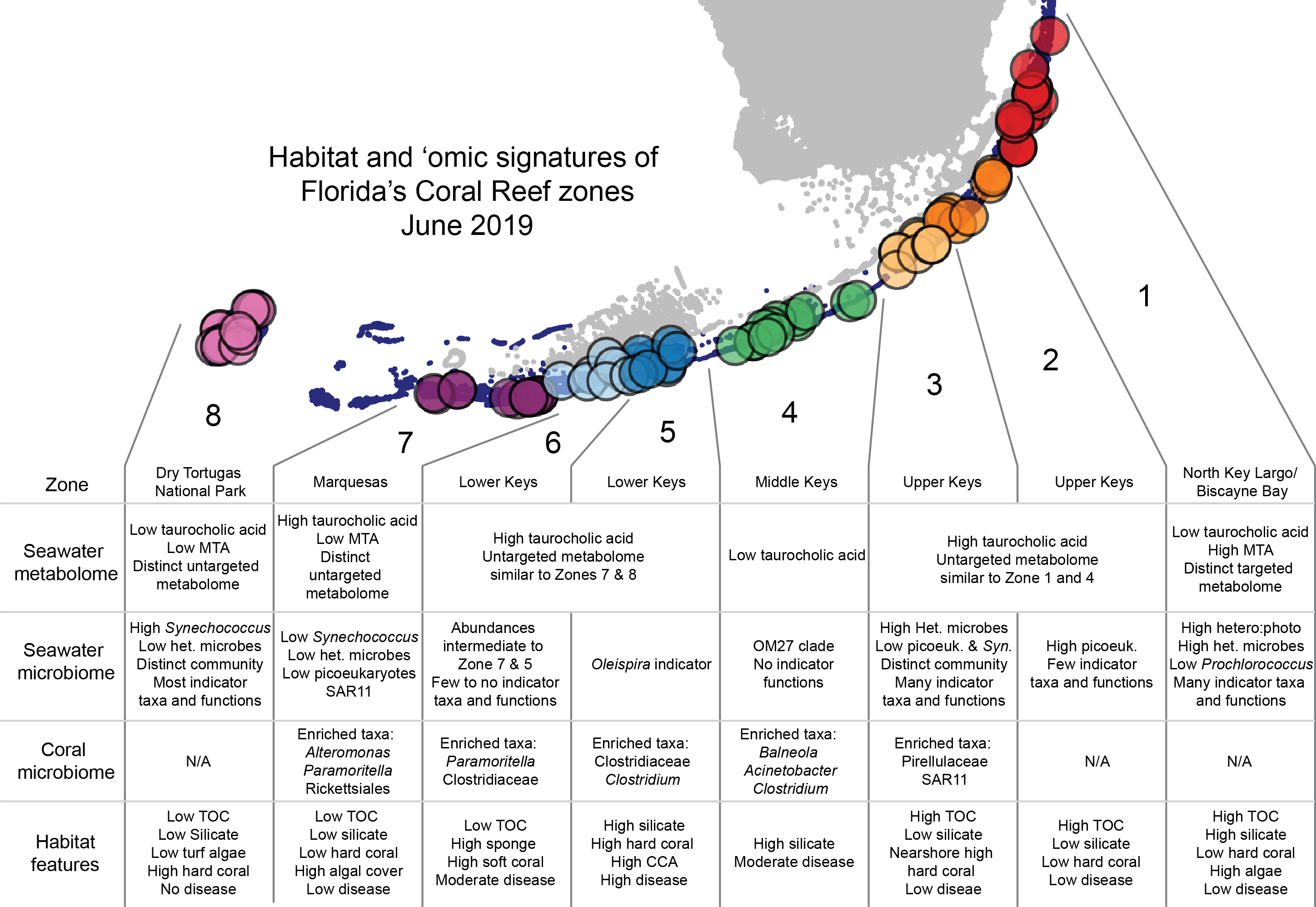


**Figure S10**. Signatures of individual Florida’s Coral Reef zones determined through water sampling for inorganic and organic nutrients, photomosaic analysis of benthic cover, metabolomic, metagenomic, and microbiome analyses within seawater and coral hosts. High and low refers to values relative to other reef zones. N/A indicates data are not available. MTA = 5’-methylthioadenosine, het. microbes = heterotrophic microbes, hetero:photo = heterotrophic microbes: photosynthetic microbes, TOC = total organic carbon, CCA = crustose coralline algae.


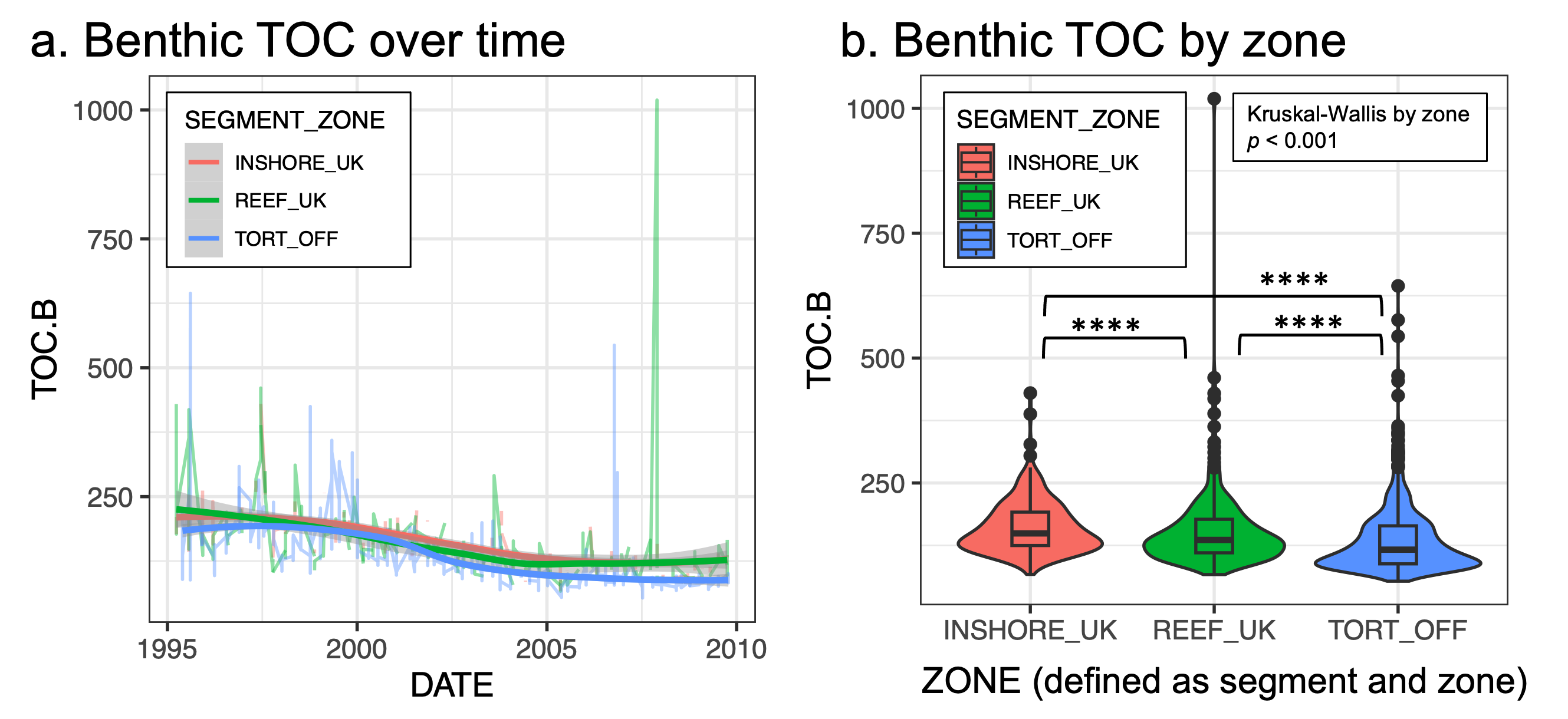


Figure S11. Concentration of total organic carbon (TOC) from benthic depths is lower at Dry Tortugas National Park area over time. A) Concentration (uM) of benthic TOC is displayed overtime, with a loess curve fitted through the data to show general trends between three regions: TORT_OFF, which refers to offshore waters within and near the boundary of Dry Tortugas National Park, INSHORE_UK and REEF_UK which refers to nearshore and above reef water samples from the Upper Keys. The Upper Keys zones match best to the Zones 1 and 2 defined in the present study. The Dry Tortugas zone matches the Zone 8 from the present study. B) Violin plots with a box and whisker plot inset show the distribution of TOC concentrations, which are significantly different across zones (Kruskal-Wallis followed by Dunn’s post-hoc test, p < 0.001), with Dry Tortugas containing the lowest concentration of TOC. These long-term data were provided by the SERC-FIU Water Quality Monitoring Network which is supported by EPA Agreement #X7 00D02412-1 and NOAA Agreement #NA09NOS4260253, and obtained through <http://serc.fiu.edu/wqmnetwork/FKNMS-CD/DataDL.htm>. Samples were collected in discrete samples using methods reported previously (1)“

References

1. H. O. Briceño, J. N. Boyer, “2017 Annual Report of the Water Quality Monitoring Project for the Water Quality Protection Program of the Florida Keys National Marine Sanctuary” (2018).
