# Supplementary material for: Microorganisms and dissolved metabolites distinguish Florida's Coral Reef habitats
Source: PNAS Nexus. 2023 Sep 5;2(9):pgad287. doi: 10.1093/pnasnexus/pgad287 (PMC10504872; doi:10.1093/pnasnexus/pgad287)
Supplement: pgad287_Supplementary_Data [file pgad287_supplementary_data.zip › PNASNEXUS-PNASNEXUS-2023-00274R-s02.docx]

***Supplementary Information - Materials and Methods for***

**Microorganisms and dissolved metabolites distinguish Florida’s Coral Reef habitats**

Cynthia C. Becker^1,2^, Laura Weber^1^, Brian Zgliczynski^3^, Chris Sullivan^3^, Stuart Sandin^3^, Erinn Muller^4,5^, Abigail S. Clark^4,6^, Melissa C. Kido Soule^1^, Krista Longnecker^1^, Elizabeth B. Kujawinski^1^, Amy Apprill^1^

^1^Marine Chemistry & Geochemistry Department, Woods Hole Oceanographic Institution, Woods Hole, MA 02543, USA

^2^MIT-WHOI Joint Program in Oceanography/Applied Ocean Science & Engineering, Cambridge, MA and Woods Hole, MA, USA

^3^Scripps Institution of Oceanography, University of California San Diego, La Jolla, CA 92093, USA

^4^Elizabeth Moore International Center for Coral Reef Research and Restoration, Mote Marine Laboratory, Summerland Key, FL 33042, USA

^5^Mote Marine Laboratory, Sarasota, FL 34236, USA

^6^The College of the Florida Keys, Key West, FL 33040, USA

**SI Methods**

*1. Study Area.* We sampled coral reef environments during a research cruise aboard the M/V *Alucia* between June 3 – 20, 2019. During this time, we conducted surveys and sampled biogeochemical seawater parameters at 85 reefs across 8 zones in Florida’s Coral Reef (FCR), from the North Key Largo/Biscayne Bay area, designated as Zone 1, to the Dry Tortugas National Park (Zone 8) (Figure 1, Supplementary File 1). We selected reefs based on input from the Florida Fish and Wildlife Conservation Commission, Florida Department of Environmental Protection, National Park Service, NOAA Coral Reef Conservation Program and Mote Marine Laboratory, with a focus on reefs that were part of long-term monitoring programs (e.g., Coral Reef Evaluation and Monitoring Project, CREMP).

The sampling design was created to ensure reefs were captured within 8 zones that matched historical reef management areas. Zone 8 was defined as reefs within the Dry Tortugas National Park. Zone 7 was defined as reefs west of Key West and near Marquesas Key. Zones 6 and 5 were chosen as they aligned with the Lower Keys, between Big Pine Key and Key West. The split between the zones was at the American Shoal Lighthouse. Zone 4 was aligned with the Middle Keys between the seven-mile bridge, Marathon Key and Long Key. Zones 3 and 2 encompassed the region of the Upper Keys between Islamorada and Key Largo that was on the opposite side of the shallower Florida Bay. The split between the zones was chosen at Plantation Key. Finally, Zone 1 extended from North Key Largo and Biscayne National Park. In the case of both the Upper and Lower Keys, these regions were split due to higher numbers of sites sampled within these geographic regions, which allowed for the potential capture of finer-scale variability across those regions. In general, there is some mismatch between our zones and those that have been adopted as biogeochemical subdivisions due to the nature of the sampling design we employed and we additionally wanted to keep Dry Tortugas separate from the offshore Keys regions due to the lack of the disease outbreak at Dry Tortugas during the time we sampled (1).

*2. Sample Collection and Ship-board Processing.* We conducted diver-based surveys to evaluate the prevalence of stony coral tissue loss disease (SCTLD) at each of the 85 reefs (Figure 1, Supplementary File 1). At each reef, one diver performed a 30-minute roving diver survey to determine the richness of scleractinian species, the presence or absence of stony coral tissue loss disease (SCTLD), and the size of all observed coral colonies. The diver assigned coral colonies to four size classes, based on diameter/length: <10cm, 10–25cm, 25–50cm, >50cm. Each diver estimated the area (m^2^) surveyed. Prevalence of SCTLD was calculated as the percent of all coral colonies exhibiting disease symptoms.

Large area imagery for benthic surveys was collected in plots (100m^2^) at 45 individual reefs using the protocol established in Edwards et. al (2)(Supplementary File 1). Three dimensional model processing was performed using Agisoft Metashape (formerly Agisoft Photoscan Pro) and Viscore as described previously in Fox et al (3).

We collected discrete seawater samples at all 85 reefs to measure inorganic nutrient (phosphate, ammonium, silicate, nitrite plus nitrate) concentrations, total organic carbon (TOC) and total nitrogen (TN) concentrations, and cell abundances (heterotrophic microbes [unpigmented bacteria and archaea], *Prochlorococcus, Synechococcus,* and picoeukaryotes) (Supplementary File 1). We collected samples via SCUBA with acid-washed and combusted 40 ml borosilicate glass vials for TOC and TN collections and 30 ml acid-washed square bottles (HDPE, Nalgene, ThermoFisher Scientific, Waltham, MA, USA) for nutrient collections, and filled both vials while at reef depth. Samples were kept on ice in a cooler for less than 4 hr prior to processing. Once on board the M/V *Alucia*, we processed all samples. We added 75 μl phosphoric acid to the 40 ml glass vials to fix the samples for TOC and TN and kept these samples at room temperature or 4°C until laboratory analysis. We removed 1.4 ml seawater from the nutrient bottles, mixed the seawater with 8% paraformaldehyde (1% final concentration, Electron Microscopy Sciences), fixed it in the dark for 20 minutes at 4°C, then froze it at −80°C. We capped the 30 ml inorganic nutrient bottles and placed them at −80°C until analysis.

We collected seawater for targeted and untargeted metabolomic analyses at 13 reefs across the 8 zones of FCR (summarized in Supplementary File 1). At each reef, we collected seawater in 1.7 l Niskin bottles via SCUBA at three distinct locations on the reef for biological replication. These Niskin bottles were kept in a cooler for less than 4 hr prior to processing on the M/V *Alucia*. Once back on board the M/V *Alucia*, we transferred seawater from the Niskin bottles into acid-washed 2l polycarbonate bottles using acid-washed PharMed BPT tubing (Masterflex, Cole-Parmer, Vernon Hills, IL, USA). These water samples were processed as described previously by Weber et al. (4). Briefly, we prefiltered the seawater through a 47 mm 0.1 μm pore size polytetrafluoroethylene filter (Omnipore, EMD Millipore Corporation, Billerica, MA, USA) to remove all microbial biomass via peristalsis and placed the filtered seawater directly into a second acid-washed 2 l polycarbonate bottle. We acidified this filtrate with 2 ml OPTIMA-grade 12 M hydrochloric acid to reach a pH of 2-3 prior to solid phase extraction (SPE). We used SPE to concentrate metabolites (primarily low molecular weight dissolved organic matter) from the filtered seawater. We used a Waters vacuum manifold to slowly pass the seawater through 1 g/6 cc SPE cartridges (Bond Elut PPL; Agilent, Santa Clara, CA, United States) pre-conditioned with HPLC-grade methanol and weighed bottles with seawater prior to and following SPE to calculate the volume of seawater filtered. SPE cartridges were wrapped in combusted aluminum foil and frozen to −80°C prior to analysis at the Woods Hole Oceanographic Institution.

We collected seawater for microbial biomass and chlorophyll analysis at 27 reefs across the 8 zones of FCR (summarized in Supplementary File 1). At each reef, we employed a groundwater pump (Mini-Monsoon 12V, Proactive Environmental Products, Bradenton, Florida, USA) to pump seawater from just above the reef benthos into acid-washed or 10% bleach-rinsed 4 l LDPE bottles (Nalgene). Samples were kept in a cooler on ice until processing less than 4 hr following collection. Once back on board the M/V *Alucia*, we used peristalsis to filter 2 l of seawater to obtain duplicates from each reef through a 0.2 μm Supor filter (Pall, Port Washington, New York, USA) for microbial biomass housed in a 25 mm filter holder (Swinnex-25, Millipore Corporation), as described previously (5). Chlorophyll samples were obtained by filtering 2 l of seawater in duplicate with the same peristalsis setup but using a GF/F filter. We placed filters (GF/F or 0.2 μm) into 2 ml cryovials and froze them at −80°C prior to further processing at the Woods Hole Oceanographic Institution.

On reefs with active stony coral tissue loss disease, we collected coral tissue and near-coral seawater samples from apparently healthy and actively diseased coral colonies. We aimed to only sample reefs where at least three healthy colonies were present in addition to at least three diseased colonies for reef-level replication. We identified 11 reefs across Zones 3-7 that met the above criteria and collected coral tissue samples from the following species: *Colpophyllia natans, Dichocoenia stokesii, Montastraea cavernosa, Orbicella faveolata,* and *Pseudodiploria strigosa.* Corals were sampled as found, with some apparently healthy colonies targeted first, and some diseased colonies targeted first, depending on reef conditions and presence of suitable colonies to sample. For each colony, collection proceeded on near-coral seawater followed by coral tissue as described previously (6). We collected near-coral seawater via 60-ml Luer-Lok^TM^ syringes within 1-5 cm of the lesion margin on colonies with active SCTLD or 1-5 cm above apparently healthy colonies. Following the near-coral seawater collections, we collected tissue from colonies with active SCTLD and from apparently healthy colonies using 10 ml Luer-Slip syringes. We collected tissue samples from along the lesion margin (i.e., between the apparently unaffected tissue and the bleached and sloughing tissue) of diseased colonies. On apparently healthy colonies (i.e., colonies without any indication of disease or other affliction), an area of the apparently healthy tissue was sampled at random. Syringes were immediately placed into individual Whirl-Pak bags to contain any mucus and tissue leaking from the syringe. Following the collections, all tissue and near-coral seawater syringe samples were placed on ice prior to processing. We transferred samples of coral tissue and mucus into 15 ml conical tubes that were transferred from ice into storage at −80°C until analysis. We attached filter holders containing 25 mm 0.2 μm Supor filters to the 60 ml Luer-Lok^TM^ syringes and depressed them by hand to capture microbial biomass on the filters. We placed the filters into labeled 2 ml cryovials and transferred them to a −80°C freezer until analysis.

*3. Benthic reef composition analysis*. Benthic composition analysis was conducted using the imagery generated from 45 reefs. We followed the Viscore and Visual Point Intercept methods with the exception that an additional 500 points were sampled per plot, resulting in 2500 stratified random points per plot, similar to methods previously reported (3). Each point was designated to highest taxonomic resolution, with points landing on coral designated to genus. Points were aggregated into 14 functional groups for benthic composition analysis of each plot. The percent out of the total points of each functional group was calculated to generate a table of benthic percent cover that was used for later analyses into reef benthic composition. We additionally subsetted the hard coral functional group for two analyses. First, we calculated the diversity metric, species richness, on hard coral by counting the number of unique species recorded at each reef. Second, we generated a table of percent cover of individual hard coral species across each reef for later analyses of hard coral composition.

*4. Flow cytometry, organic nutrient, inorganic nutrient, and chlorophyll analyses for water quality.* Flow cytometry samples were processed and analyzed by the University of Hawaii SOEST Flow Cytometry Facility as described previously (5). Briefly, each sample was stained with Hoechst 33342 DNA stain and excited with both 488 nm (1W) and UV (~350 nm, 200 mW) lasers co-linearly on a Beckman-Coulter Altra flow cytometer (Beckman Coulter Life Sciences). Signals of forward and side scatter and fluorescence were analyzed to distinguish populations and abundances (cells ml^−1^) of four cell types: *Prochlorococcus, Synechococcus,* eukaryotic picophytoplankton (picoeukaryotes), and non-pigmented bacteria. Non-pigmented prokaryotes were used as a proxy for heterotrophic bacterial and archaeal cells (7, 8), and are referred to as “heterotrophic microbes” in the manuscript.

Non-purgeable total organic carbon (TOC) samples and total nitrogen (TN) samples were analyzed with a Shimadzu TOC-V_CSH_ TOC analyzer (9) using a TNM-1 module. We shipped inorganic nutrient samples to Oregon State University for analysis of phosphate, ammonium, silicate, nitrite and nitrate, as in Apprill and Rappé (10). Briefly, samples were run on a Technicon AutoAnalyzer II (SEAL Analytical) and an Alpkem RFA 300 Rapid Flow Analyzer to generate nutrient concentrations (μM). We determined total organic nitrogen (TON) concentrations by subtracting concentrations of inorganic nitrogen (ammonium and nitrite plus nitrate) from total nitrogen.

Chlorophyll was extracted with acetone using standard methods (11). Filters were thawed individually and immediately placed in a glass test tube with 5 ml or 10 ml of 90% acetone, with 10 ml used in the case the filter appeared particularly dark, and capped. The filters were left to extract for 24 hours in the dark at 4°C. After the extraction, the tubes were vortexed and centrifuged to concentrate any particulate matter at the bottom of the tube. Prior to analysis, blanks including air, 90% acetone, and a black standard were run on an AquaFluor fluorometer (Turner Designs handheld 800446) fitted with a red sensitive photomultiplier. Approximately 3 ml of solvent was analyzed on the fluorometer at wavelength of 664 nm, followed by acidifying the sample with two drops of 10% hydrochloric acid, then measuring again to assess phaeopigment concentration. Readings were corrected for the volume filtered and concentration of chlorophyll was measured by referencing a standard curve.

*5. Targeted and untargeted metabolomic laboratory processing and mass spectrometry*. We eluted dissolved organic matter (DOM) from the SPE cartridges and prepared samples for analyses as outlined by Weber and colleagues (4). To summarize, 4 bed-volumes of 0.01 M HCl were added to the cartridges to remove salt. The cartridges were then dried for five minutes and eluted into combusted glass vials using 6 ml of 100% methanol. Extracts were frozen at –20ºC until they were dried down using a vacuum centrifuge. Extracts were then resuspended with a 95:5 (v/v) MilliQ water: acetonitrile (ACN) solution with deuterated biotin (final concentration 0.05 mg ml^−1^) (200 μl total) and vortexed. A pooled sample was made for all mass spectrometry runs by combining equal volume aliquots from all extracts into one vial. The pooled sample was injected throughout both analytical runs to monitor instrument drift and run quality. After preparation, all extracts were stored at −20ºC until analysis. For targeted metabolomics, 100 μl aliquots of each extract were placed in separate vials with combusted glass inserts. For the untargeted metabolomics analysis, 300 μl of the deuterated biotin standard and water: ACN solution was used to dilute a 25 µl aliquot of each extract. Untargeted metabolite analysis was performed using an ultrahigh performance liquid chromatography system (Vanquish UHPLC, Thermo ScientificTM) coupled with an Orbitrap Fusion Lumos Tribid mass spectrometer (Thermo ScientificTM). A Vanguard pre-column and Waters Acquity HSS T3 column (2.1 mm × 100 mm, 1.8 μm), was used for chromatographic separation at 40ºC. The column was eluted at 0.5 ml min^−1^ with the following solvents: A) 0.1% formic acid in water and B) 0.1% formic acid in ACN. The chromatographic gradient was: 1% B for 1 min, 15% B for 1 – 3 min, 50% B for 3 – 6 min, 95% B for 6 – 9 min, and 95% B for 10 min. Between injections, the column was washed and re-equilibrated with 1% B for 2 min. Individual autosampler injections (5 μl each) were made for negative and positive ion mode analyses. In negative ion mode, the electrospray voltage was set to 2600 V. Settings for source gases were 55 (sheath), 20 (auxiliary), and 1 (sweep) in arbitrary units. The temperatures of the heated capillary and vaporizer were 350ºC and 400ºC, respectively. MS data were collected in the Orbitrap analyzer with a mass resolution of 120,000 FWHM at *m/z* 200. The automatic gain control (AGC) target was 4e5, with a 50 sec maximum injection time, and a scan range of 100 – 1000 *m/z*. Data dependent MS/MS spectra were collected at 7,500 resolution in the Orbitrap analyzer. Parent ions were isolated with a 1 *m/z* width in the quadrupole, and fragmented with a HCD (higher energy collisional dissociation) energy of 35%. All data were collected in profile mode. Samples were run in a random order and after every seven samples, a pooled sample was run. Raw data files from the instrument were converted into mzML files using msConvert and then processed using XCMS (12, 13). Peak-picking was performed with the CentWave algorithm and a Gaussian fit with the following parameters: noise = 10000, peak-width = 3 – 15, ppm = 15, prefilter = c(2,168.600), integrate = 2, mzdiff = −0.005, snthresh = 10. Retention times were then adjusted using Orbiwarp and correspondence between the peaks was conducted. The coefficient of variation across the eight untargeted pooled sample features was 0.044, demonstrating good agreement between the pooled samples, and the pooled samples were removed from further analyses. In the untargeted analysis, only MS1 features were analyzed, and were defined as unique combinations of mass-to-charge ratios (*m/z*) and retention times (RT). This analysis yielded a table of MS1 features (*m/z* x RT) and their peak intensities across each sample.

Extracts prepared for targeted metabolomics were run on a triple stage quadrupole mass spectrometer (TSQ Vantage, Thermo Fisher Scientific^TM^) using UHPLC (Accela Open Autosampler and Accela 1250 Pump, Thermo Scientific^TM^) coupled to a heated electrospray ionization source (H-ESI) and operated in selective reaction monitoring (SRM) mode. The same chromatography column, conditions, gradient, and flow rates were used for targeted analyses as those described for untargeted analyses. Separate autosampler injections of 5 μl each were made for positive and negative modes. Additionally, as with the untargeted analysis, samples were run in a random order and pooled samples were run every seven samples. SRM parameters were optimized for each compound using a standard as described in Kido Soule et al. (14) and two SRM transitions (precursor – product ion pairs) were monitored for quantification and confirmation. Target metabolites included compounds found in central carbon metabolism and metabolites that are environmentally relevant in marine habitats or are produced by marine microorganisms (14–16). The resulting XCalibur raw files (MS/MS data) were converted into mzML files using msConvert (13) and processed with the open-source program El-MAVEN (v.774)(17). Using El-MAVEN, 8-point calibration curves based on integrated peak area were generated for each compound. Environmental concentrations of metabolites were determined by dividing each concentration by the original sample collection volume. Next, metabolites that passed the limits of detection and quantification for the UPLC-MS/MS analysis (Kido Soule, Longnecker, Swarr, unpublished) were corrected for extraction efficiency based on published data for each metabolite in seawater (18).

To prepare the untargeted metabolomic data for statistical analyses, data from mass spectrometry runs in both positive and negative ion modes were filtered and normalized using blank correction, low coefficient of variation filtration, and seawater volume normalization. First, for the blank correction, metabolite features were kept if they exhibited an average fold change greater than 1 across all samples compared to the blanks. This removed 11% and 9% of metabolite features from negative and positive ion mode data, respectively. Second, metabolite peak intensities with a low coefficient of variation (CV) across samples were removed to maintain only the metabolites with more variable peak intensities. Overall, the CV of metabolite peak intensities was low across samples so a cutoff at the third quartile removed metabolites with a CV below 0.081 (Negative mode) or 0.079 (Positive mode). The remaining peak intensities from metabolite features (1,428 from negative mode and 2,759 from positive mode) were normalized to volume of seawater. These procedures yielded tables of filtered MS1 features (*m/z* x retention time) and their normalized peak intensities across each sample.

*6. DNA extraction and sequencing for 16S rRNA and shotgun metagenomes*

We extracted DNA from 25 mm filters used for 2 l seawater collections using Qiagen PowerBiofilm kits (Qiagen, Germantown, MD, USA). To begin, we added the filter directly to the bead tube, then proceeded with the extraction following manufacturer protocols. We also included four DNA extraction controls by extracting DNA from unused filters. Resulting DNA from these extractions were used as the template for both 16S rRNA gene sequencing and shotgun metagenomic sequencing.

For 16S rRNA gene sequencing of bacteria and archaea, we included 2 μl of template DNA into a 50 μl (total volume) PCR reaction. We added a PCR negative control by including one PCR reaction with 2 μl of PCR grade H_2_O instead of DNA. Earth Microbiome Project primers, 515F (19) and 806R (20), were used to amplify the V4 region of the small subunit (SSU) rRNA gene in bacteria and archaea and included sample-specific barcodes with an 8 bp barcode, 10 bp pad, and 2 bp link, similar to Kozich et al. (21). The 50 μl reactions were diluted in UV-sterilized nuclease-free water and contained 2.5 units of GoTaq DNA Polymerase (Promega, Madison, WI, USA), barcoded primers at 0.2 μM, 0.2 mM dNTP mix (Promega), 2.5 mM MgCl_2_, and 1X colorless GoTaq flexi buffer (Promega). The reactions were run on a Bio-Rad Thermocycler using the following criteria: denaturation at 95°C for 2 min; 28 cycles at 95°C for 20 s, 55°C for 15 s, and 72°C for 5 min; and extension at 72°C for 10 min. We used gel electrophoresis to verify successful amplification using 5 μl of product on a 1% agarose-Tris-borate-EDTA (TBE) gel stained with SYBR Safe gel stain (Invitrogen, ThermoFisher Scientific). We used the QIAquick 96 PCR Purification Kit (Qiagen) with the QIAvac 96 (Qiagen) and vacuum pressure to purify the remaining 45 μl of PCR products following manufacturer’s protocols. We applied the HS dsDNA assay on the Qubit 2.0 fluorometer (ThermoFisher Scientific) to quantify the DNA concentrations then converted to nM assuming an average library size of 450 bp, and average molar mass of DNA nucleotides of 660 g/mol. We diluted individual barcoded PCR products to 10 nM, pooled all samples, and shipped the pooled, ready-to-run library to the Georgia Genomics and Bioinformatics Core at the University of Georgia for sequencing on an Illumina MiSeq using paired-end 250 bp sequencing.

We prepared a library for shotgun metagenomic sequencing following the Illumina DNA Prep Reference Guide (Illumina, San Diego, CA, USA, Document # 1000000025416 v09 June 2020). DNA input for all samples was between 100-500 ng. Concentrations of the four DNA extraction control samples were below detection, so we included 30 ul of each in the procedure and processed them in the same way as all seawater samples. We used IDT for Illumina DNA/RNA UD Indexes Set A, Tagmentation (Illumina, 96 samples, Cat # 20027213), to apply sample-specific indices. Following the procedure, we eluted samples in 30 μl resuspension buffer. To verify successful processing, we used a fluorometric assay (HS dsDNA) on a Qubit 2.0 fluorometer to measure DNA concentrations of a subset of samples. All final concentrations were greater than 4 ng/μl, and therefore deemed sufficient for pooling. All samples were pooled, and the final concentration was 5.30 ng/μl. The final library was run at the Georgia Genomics and Bioinformatics Core at the University of Georgia on an Illumina NextSeq 2000 with the P3 flow cell and paired-end 150 bp sequencing.

*7. On-ship near-coral seawater sample processing and sequencing*

To expedite the turnaround time between sample collection and sample processing, we performed on-ship DNA extraction, PCR, and sequencing with the Illumina iSeq 100 System on near-coral seawater samples following methods for in-the-field microbiome preparation described previously (6). Seawater samples targeted for on-ship sequencing included those sampled from 5 reefs over two days (June 9-10, 2019).

*8. DNA extraction and sequencing of near-coral seawater and coral microbiomes*

All processing of near-coral seawater and coral tissue slurries proceeded as described previously to identify bacteria and archaea within each environment (6). Briefly, we extracted DNA from all seawater and tissue samples using the DNeasy PowerBiofilm kit (Qiagen). PCR occurred in a two-stage procedure. In stage one, we used Earth Microbiome Project primers, 515F (19) and 806R (20), to amplify the V4 region of the small subunit (SSU) ribosomal RNA gene of bacteria and archaea*.* In stage two, we attached unique index primers to each sample using the Nextera XT v2 set A kit (Illumina). For PCR that occurred at the Woods Hole Oceanographic Institution, we used larger benchtop centrifuges (Eppendorf 5418) and thermocyclers (Bio-Rad), rather than the small and portable versions used on the M/V *Alucia*. Following purification of stage two PCR products, we diluted and pooled samples such that we included approximately 40 samples. Seawater and tissue samples were randomized across all library pools. Pooled libraries were diluted to approximately 90 pM, and a 10% PhiX Control v3 (Illumina) spike-in was added to increase base diversity. All libraries were run on the Illumina iSeq 100 System (Illumina) with the i1 cartridge pack, over a total of 6 sequencing runs.

*9. Benthic survey, disease survey, and water quality data analysis.*

Benthic cover, organic and inorganic nutrients, cell abundances, hard coral species richness, and disease prevalence data were measured from 45 reefs (benthic cover, hard coral species richness) or 85 reefs (all other parameters) in FCR across eight zones. To evaluate which environmental parameters changed significantly across reef zones, we conducted a Kruskal-Wallis test in R (v4.0.3) and evaluated significance of the results against a Bonferroni-corrected p-value to account for multiple comparisons (Figure 2). For significant environmental variables, we followed up with a pairwise Wilcoxon rank sum test to investigate which zones were significantly different from other zones at a p-value < 0.05 after a Benjamini-Hochberg false discovery rate adjustment (Supplementary File 2).

To examine the changes in benthic and hard coral composition across FCR, we conducted a principal component analysis (PCA) in R and visualized the PCA using the function “fviz_pca” and displayed the strength of each benthic component with the PCA. We used the package *vegan* and function adonis() to conduct a PERMANOVA test by zone using the formula adonis(benthic_data ~ zone, data = metadata, method = “eu”).

To identify environmental conditions that were significantly different at Dry Tortugas National Park (Zone 8) compared to all other reefs and zones in FCR combined, we conducted a Wilcoxon Rank Sum test and the results were deemed significant at a Bonferroni corrected p-value less than 0.001667 (Figure S3). Additional linear regressions were conducted to individually evaluate the influence the relationship between hard coral cover and algal cover using lmodel2(algae ~ log(Hard coral)) (Figure S7), and the effect of distance to shore on hard coral cover using lm(hard coral ~ distance to shore) (Figure S6). For the relationship between algae and hard coral cover, a model II is regression was used as both variables contain sources of variation. Standard major axis (SMA) model II regressions were used specifically after evaluating the significance of the correlation coefficient (r) and because both variables were normally distributed, but had different units as hard coral cover had to be log transformed to satisfy assumptions of normality.

*10. Targeted Metabolomics analysis.*

Targeted metabolites were corrected for extraction efficiency and converted to picomolar (pM) concentrations prior to analysis (see above). The metabolites were grouped by Zone, except the two zones in the upper keys (Zones 2 & 3) and the zones in the lower keys (Zone 5 & 6) that were combined to ensure at least two reefs (n = 3 per reef) were in each group for statistical tests. To evaluate how the 39 quantified metabolites related to the environmental conditions in FCR, we conducted a distance-based redundancy analysis (dbRDA) using the *vegan* (v2.5-7) package (22). Further dbRDA details are below. The gower distance metric for the dbRDA was chosen because it yielded the highest value after comparing several distance metrics with the “rankindex” function in the *vegan* R package. As with the environmental parameters, we identified targeted metabolites that significantly changed with zone by conducting a Kruskal-Wallis test in R (v4.0.3) and evaluated significance of the results against a Bonferroni-corrected p-value to account for multiple comparisons (Figure 3). For significant metabolites, we followed up with a pairwise Wilcoxon rank sum test to investigate which zones were significantly different from other zones at a p-value < 0.05 after a Benjamini-Hochberg false discovery rate adjustment (Supplementary File 2).

*11. 16S rRNA sequence analysis to generate amplicon sequence variants.*

Sequence reads from the Illumina MiSeq run were inspected for quality, trimmed, filtered, and amplicon sequence variants (ASVs) were generated using the *DADA2* R package (v1.18.0) (23). We used default filtering parameters and trimming parameters were trimLeft = c(20,20) and truncLen=c(240,200) based on when quality profiles began to drop below a quality score of around 30. For data generated by the Illumina iSeq (near coral seawater and coral microbiomes), we used the parameters trimLeft=20 and truncLen=125 and only forward reads were used due to lack of overlap between forward and reverse reads. For MiSeq-generated data, we also merged forward and reverse reads, removed chimeras and assigned the taxonomy of ASVs in DADA2. For taxonomic assignment, we used the SILVA SSU rRNA database (v138) and assigned species, when possible (24). We removed any ASVs that classified as mitochondria and chloroplasts, and filtered out ASVs with an average count of 0.5 across samples to remove low abundant taxa. We also removed DNA extraction controls and PCR controls for data analysis. Additionally, ASVs were given a unique number identifier for tractability in the manuscript. The associated DNA sequences are published in Supplementary File 3. This ASV generation and filtering from *DADA2* yielded two tables of ASVs (one for seawater microbiomes from MiSeq sequencing and one for near-coral seawater and coral microbiomes from the iSeq) and their counts across samples were transformed to relative abundances and log transformed after a pseudo count addition of 1 for further data analysis.

*12. Shotgun metagenomic analysis to generate bacterial functional data.*

Shotgun sequencing on the Illumina NextSeq yielded 13,592,781.7 ± 3,357,010.1 paired-end sequence reads per reef seawater microbial community sample. Due to sequencing errors, 6 of the reef samples had single samples, while the other 21 had duplicate sequence samples. We inspected read quality using FastQC (0.11.9) running default parameters. FastQC results showed high quality sequences. Based on the FastQC output, we trimmed forward and reverse reads with Trimmomatic (v0.39) using the parameters “SLIDINGWINDOW:4:20” and “MINLEN:50” to trim reads if quality dropped below 25 over 4 bases and to remove any reads with fewer than 50 bp, respectively. This retained approximately 94.16% ± 0.37% of sequence reads per sample. Trimmed paired-end reads were then used as input for a metagenome co-assembly of all samples using MegaHit (v1.2.9)(25). We chose MegaHit as the assembler as it has been shown to generate more genes that could be successfully annotated compared to MetaSPAdes in complex environments such as ocean and soil samples (20). MegaHit parameters included --min-contig-len 500 --continue -t 60. We further filtered the output metagenome assembly using seqkit (v0.16.1) to retain only contigs greater than 1000 bp. We used Quast (v5.0.2) to measure assembly statistics. The filtered metagenome assembly had an N50 of 2,742 and contained 1,041,682 contigs.

To obtain functional annotations of the metagenome assembly, we used Prokka (v1.14.6). With the functional annotations, the .ffn file containing nucleotide sequences of each annotated gene was input into Salmon (v3.7.4) for read mapping of short reads to the functional annotations to generate abundances of each functional gene at each reef. This yielded a table of sequence counts of each annotated gene across the 27 samples. We retained the 592,973 genes with Clusters of Orthologous Genes (COG) identifiers. We additionally included pathway information for the relevant COG IDs by downloading the latest COG database FTP from <https://ftp.ncbi.nih.gov/pub/COG/COG2020/data/> and using the cog-20.def.tab document.

To prepare the abundance matrix for statistical analysis, we filtered out low-abundance genes with an average count of < 0.5 across the samples, transformed the gene counts to relative abundances, and log transformed after adding a pseudocount of 1.

*13. Comparative ‘omic data analysis*.

To evaluate how the composition of metabolite features, taxonomic microbial communities, and microbial functional genes changed across different reefs, we calculated Bray-Curtis dissimilarity across different reefs using the function vegdist() in *vegan* (v2.5-7) and then calculated principal coordinates with the function cmdscale() in R (22). The resulting principal coordinates were plotted using *ggplot2* (v3.3.3) (26). To test the influence of zone, Dry Tortugas versus all other zones combined, disease prevalence, and sampling date on the compositions of metabolites or microbial genomic data, we used the adonis() function in *vegan*.

A distance-based redundancy analysis (dbRDA) was calculated to evaluate the significant association between sample metadata (zone) and environmental variables (TOC, heterotrophic microbes, *Prochlorococcus, Synechococcus,* picoeukaryotes, stony coral tissue loss disease prevalence, hard coral percent cover, algae (fleshy macroalgae and turf algae) percent cover, soft coral percent cover, sponge percent cover, and hard coral richness) and the relative abundances of ASVs. We calculated the dbRDA with Bray-Curtis dissimilarity using the capscale() function in *vegan*. The model formula was as follows: “capscale(OmicsDataFrame ~ Zone + SCTLD + TOC + Het. microbes + Prochlorococcus + Synechococcus + picoeukaryotes + hard coral + algae + soft coral + sponge + hard coral richness, EnvironmentalVariables_Dataframe, distance = "bray", na.action = na.exclude). Note for the targeted metabolomics analysis, distance= “gower” was used. We evaluated the significance of the response variables (environmental variables and metadata) using an analysis of variance (ANOVA) with the parameter by = “terms”. Response variables were evaluated as significant when p < 0.05 and they were plotted onto the ordination with *ggplot2*. To examine the variance in the ‘omics data explained by the different environmental variables, we used variance partitioning using the function varpart() in *vegan.* The varpart function only takes up to four explanatory tables at once, so we used the following three varpart calls: varpart(Y = dissimilarity.matrix, X = ~zone, ~SCTLD, ~Total organic carbon, ~hard_coral_richness data = env.variables), varpart(Y = dissimilarity.matrix, X = ~het. microbes, ~prochlorococcus, ~synechococcus, ~picoeukaryotes, data = env.variables), and varpart(Y = dissimilarity.matrix, X = ~Hard_coral, ~MA_turf, ~Soft_coral, ~Sponge, data = env.variables). We reported the Adjusted R^2^ result associated with the individual fractions. In some cases the R^2^ was negative, which is due to the nature of the dbRDA, which can return negative eigenvalues.

To identify significant untargeted metabolites that changed with FCR zones, an ANOVA or Kruskal-Wallis test was used based on the normality of the data, tested with a Shapiro-Wilk test. Metabolite features in both negative and positive ion modes were assumed significant following p < 0.05 that was Bonferroni-corrected based on the number of metabolite features. The resulting 56 untargeted features were z-score standardized and visualized with a heatmap using the “heatmap.2” function in the R package *gplots* (v3.1.3).

To identify taxa and functional microbiome indicators of FCR, we used the multi-level pattern analysis, multipatt() function in the R package *indicspecies* (v1.7.12). The function implemented the “IndVal.g” function and microbiome taxa or functional genes were identified as indicators if they had a positive predictor value (A) over 0.6, sensitivity (B) over 0.6 and were significant at a p < 0.05. Tiled heatmaps were generated in *ggplot2* to visualize all indicator ASVs and functional genes, with a summary of all results presented in Supplementary File 3.

To evaluate the abundance of genes for source and sink proteins of 5’-methylthioadenosine (MTA), we manually filtered the full list of annotated genes (prior to low abundance filtering) to search for genes encoding proteins that use MTA (sinks - MTA/S-adenosylhomocysteine (SAM) deaminase, COG0402, EC 3.5.4.28; MTA/SAM nucleosidase, COG0775, EC 3.2.2.9; MTA phosphorylase, COG0005, EC 2.4.2.28) and genes encoding proteins that produce MTA (sources – polyamine aminopropyltransferase, COG0421, EC 2.5.1.16, EC 2.5.1.104; Isovaleryl-homoserine lactone synthase, COG3916, EC 2.3.1.228; S-adenosylmethionine:diacylglycerol 3-amino-3-carboxypropyl transferase, COG5379). The relative abundance of all source and sink genes encoding these proteins was added up by sample and averaged across technical duplicates. Then, a model II simple linear regression using the ordinary least squares (OLS) method was used to relate the square-root transformed concentration of MTA biological triplicates at each reef to the relative abundance (averaged over technical duplicates) of source and sink genes at that reef. The regression was computed using the *lmodel2* (v1.7.3) package in R. The OLS method was used as the MTA concentration failed normality tests (Shapiro-Wilk test p > 0.05). Permuted 1-tailed p-values were computed using 999 permutations.

*14. Near-coral seawater and coral microbiome analyses.*

Near coral seawater and coral taxonomic microbiome beta diversity was calculated with Bray-Curtis dissimilarity and visualized with principal coordinates analysis using the R packages *vegan* and *ggplot2*. The significant influence of zone, disease, and coral species on microbiome composition was tested with the adonis() function in *vegan* (v2.5-7) using 999 permutations and the resulting R^2^, F, and p-values were reported to assess the significance of the relationships. Healthy coral taxonomic microbiomes were subset for differential abundance analysis to test which ASVs significantly changed by reef zone using the R package *corncob* (27). Zone 3 was used as the reference zone as it was the most northeast region and the test was run in *corncob*, which models relative abundance of ASV raw counts with a logit-link for mean and dispersion. Differential abundance of coral ASVs were modeled as a linear function of reef zone. We tested the hypotheses that relative abundance of an ASV changed significantly with respect to reef zone using the parametric Wald test. Significantly changing ASV relative abundances were assessed at a Benjamini-Hochberg false discovery rate corrected p-value of 0.05.

**REFERENCES**

1. H. O. Briceño, J. N. Boyer, J. Castro, P. Harlem, Biogeochemical classiﬁcation of South Florida’s estuarine and coastal waters. *Marine Pollution Bulletin*, 18 (2013).

2. C. B. Edwards, *et al.*, Large-area imaging reveals biologically driven non-random spatial patterns of corals at a remote reef. *Coral Reefs* **36**, 1291–1305 (2017).

3. M. D. Fox, *et al.*, Limited coral mortality following acute thermal stress and widespread bleaching on Palmyra Atoll, central Pacific. *Coral Reefs* **38**, 701–712 (2019).

4. L. Weber, *et al.*, Extracellular Reef Metabolites Across the Protected Jardines de la Reina, Cuba Reef System. *Front. Mar. Sci.* **7**, 582161 (2020).

5. C. Becker, *et al.*, Microbial and nutrient dynamics in mangrove, reef, and seagrass waters over tidal and diurnal time scales. *Aquat Microb Ecol* **85**, 101–119 (2020).

6. C. C. Becker, M. Brandt, C. A. Miller, A. Apprill, Microbial bioindicators of Stony Coral Tissue Loss Disease identified in corals and overlying waters using a rapid field‐based sequencing approach. *Environ Microbiol*, 1462-2920.15718 (2021).

7. B. C. Monger, M. R. Landry, Flow cytometric analysis of marine bacteria with Hoechst 33342. *Appl Environ Microbiol* **59**, 905–911 (1993).

8. D. Marie, F. Partensky, S. Jacquet, D. Vaulot, Enumeration and cell cycle analysis of natural populations of marine picoplankton by flow cytometry using the nucleic acid stain SYBR green I. *Applied and Environmental Microbiology* **63**, 8 (1997).

9. D. A. Hansell, C. A. Carlson, Biogeochemistry of total organic carbon and nitrogen in the Sargasso Sea: control by convective overturn. *Deep Sea Research Part II: Topical Studies in Oceanography* **48**, 1649–1667 (2001).

10. A. Apprill, M. Rappé, Response of the microbial community to coral spawning in lagoon and reef flat environments of Hawaii, USA. *Aquat. Microb. Ecol.* **62**, 251–266 (2011).

11. JGOFS, “Protocols for the joint global ocean flux study (JGOFS) core measurements (Report 19)” (IOC SCOR, 1996).

12. C. A. Smith, E. J. Want, G. O’Maille, R. Abagyan, G. Siuzdak, XCMS: Processing Mass Spectrometry Data for Metabolite Profiling Using Nonlinear Peak Alignment, Matching, and Identification. *Anal. Chem.* **78**, 779–787 (2006).

13. M. C. Chambers, *et al.*, A cross-platform toolkit for mass spectrometry and proteomics. *Nat Biotechnol* **30**, 918–920 (2012).

14. M. C. Kido Soule, K. Longnecker, W. M. Johnson, E. B. Kujawinski, Environmental metabolomics: Analytical strategies. *Marine Chemistry* **177**, 374–387 (2015).

15. C. L. Fiore, K. Longnecker, M. C. Kido Soule, E. B. Kujawinski, Release of ecologically relevant metabolites by the cyanobacterium *S* *ynechococcus elongatus* CCMP 1631: Metabolomics of *Synechococcus*. *Environ Microbiol* **17**, 3949–3963 (2015).

16. C. L. Fiore, C. J. Freeman, E. B. Kujawinski, Sponge exhalent seawater contains a unique chemical profile of dissolved organic matter. *PeerJ* **5**, e2870 (2017).

17. S. Agrawal, *et al.*, “El-MAVEN: A Fast, Robust, and User-Friendly Mass Spectrometry Data Processing Engine for Metabolomics” in *High-Throughput Metabolomics*, Methods in Molecular Biology., A. D’Alessandro, Ed. (Springer New York, 2019), pp. 301–321.

18. W. M. Johnson, M. C. Kido Soule, E. B. Kujawinski, Extraction efficiency and quantification of dissolved metabolites in targeted marine metabolomics. *Limnol. Oceanogr. Methods* **15**, 417–428 (2017).

19. A. E. Parada, D. M. Needham, J. A. Fuhrman, Every base matters: assessing small subunit rRNA primers for marine microbiomes with mock communities, time series and global field samples. *Environ Microbiol* **18**, 1403–1414 (2016).

20. A. Apprill, S. McNally, R. Parsons, L. Weber, Minor revision to V4 region SSU rRNA 806R gene primer greatly increases detection of SAR11 bacterioplankton. *Aquat. Microb. Ecol.* **75**, 129–137 (2015).

21. J. J. Kozich, S. L. Westcott, N. T. Baxter, S. K. Highlander, P. D. Schloss, Development of a Dual-Index Sequencing Strategy and Curation Pipeline for Analyzing Amplicon Sequence Data on the MiSeq Illumina Sequencing Platform. *Applied and Environmental Microbiology* **79**, 9 (2013).

22. J. Oksanen, *et al.*, vegan: community ecology package. *R package version 2.5-7* (2020).

23. B. J. Callahan, *et al.*, DADA2: High-resolution sample inference from Illumina amplicon data. *Nat Methods* **13**, 581–583 (2016).

24. C. Quast, *et al.*, The SILVA ribosomal RNA gene database project: improved data processing and web-based tools. *Nucleic Acids Res* **41**, D590–D596 (2012).

25. D. Li, C.-M. Liu, R. Luo, K. Sadakane, T.-W. Lam, MEGAHIT: an ultra-fast single-node solution for large and complex metagenomics assembly via succinct *de Bruijn* graph. *Bioinformatics* **31**, 1674–1676 (2015).

26. H. Wickham, *ggplot2: Elegant Graphics for Data Analysis*, 2nd ed. 2016 (Springer International Publishing : Imprint: Springer, 2016) https:/doi.org/10.1007/978-3-319-24277-4.

27. B. D. Martin, D. Witten, A. D. Willis, Modeling microbial abundances and dysbiosis with beta-binomial regression. *Ann. Appl. Stat.* **14**, 94–115 (2020).
